# Supplementary material for: Hydrogen-deuterium exchange mass spectrometry captures distinct dynamics upon substrate and inhibitor binding to a transporter
Source: Nat Commun. 2020 Dec 2;11:6162. doi: 10.1038/s41467-020-20032-3 (PMC7710758; doi:10.1038/s41467-020-20032-3)
Supplement: Supplementary file 1 — Supplementary Information [file 41467_2020_20032_MOESM1_ESM.pdf]

Hydrogen-deuterium exchange mass spectrometry captures distinct dynamics upon substrate and inhibitor binding to a transporter

Jia et al.

Supplementary Information

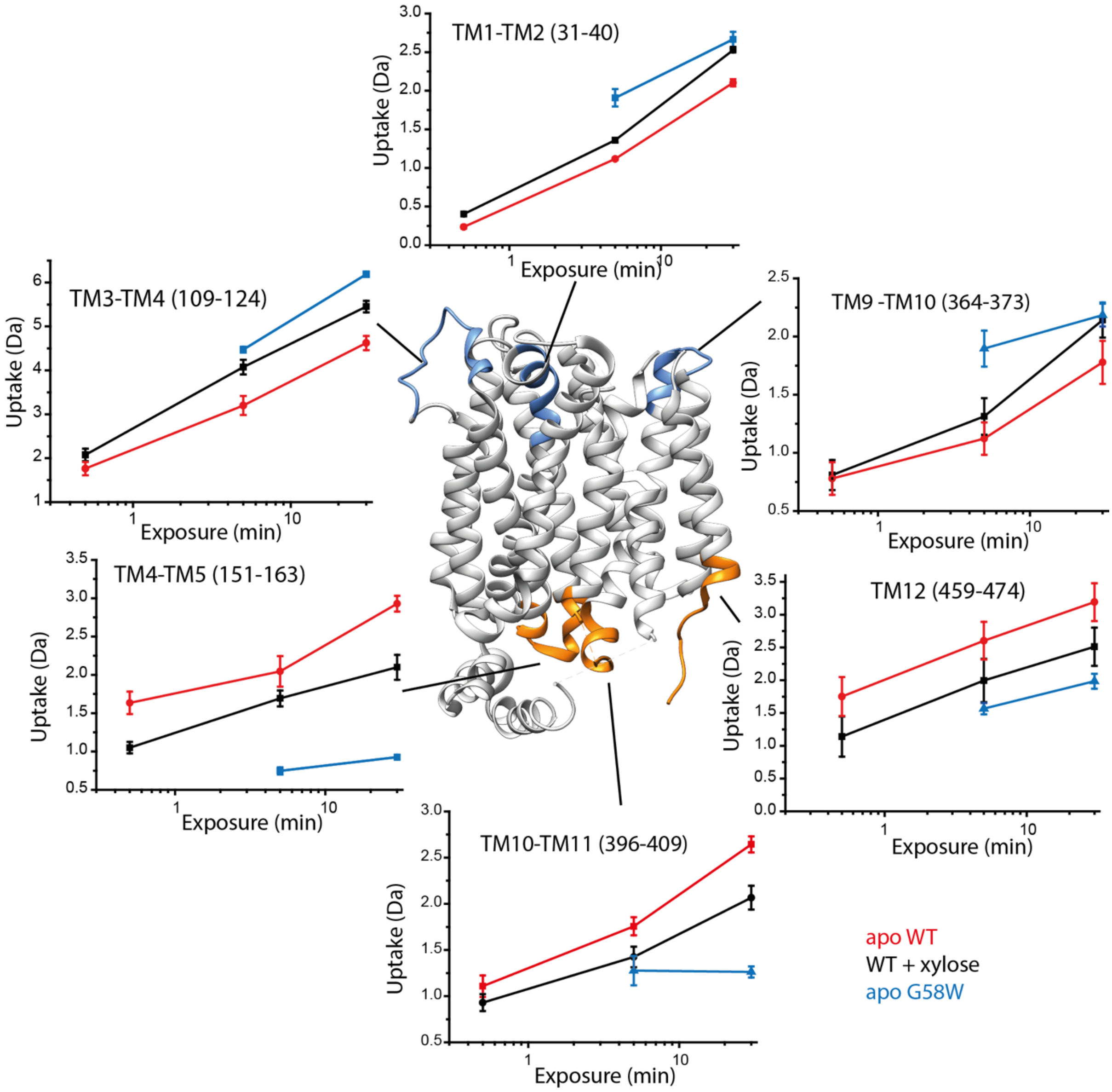

Supplementary Figure 1. Deuterium uptake plots of peptides identified as conformational reporters for XylE [1]. The regions selected as conformational reporters are based on the ΔHDX between WT apo and G58W apo. Their location on the structure is shown on the IF conformation (PDB: 4JA3) with the extracellular reporters in blue and the intracellular reporters in orange. Standard deviations for each time point are plotted as error bars (n=3).

Relative fractional uptake - replicates

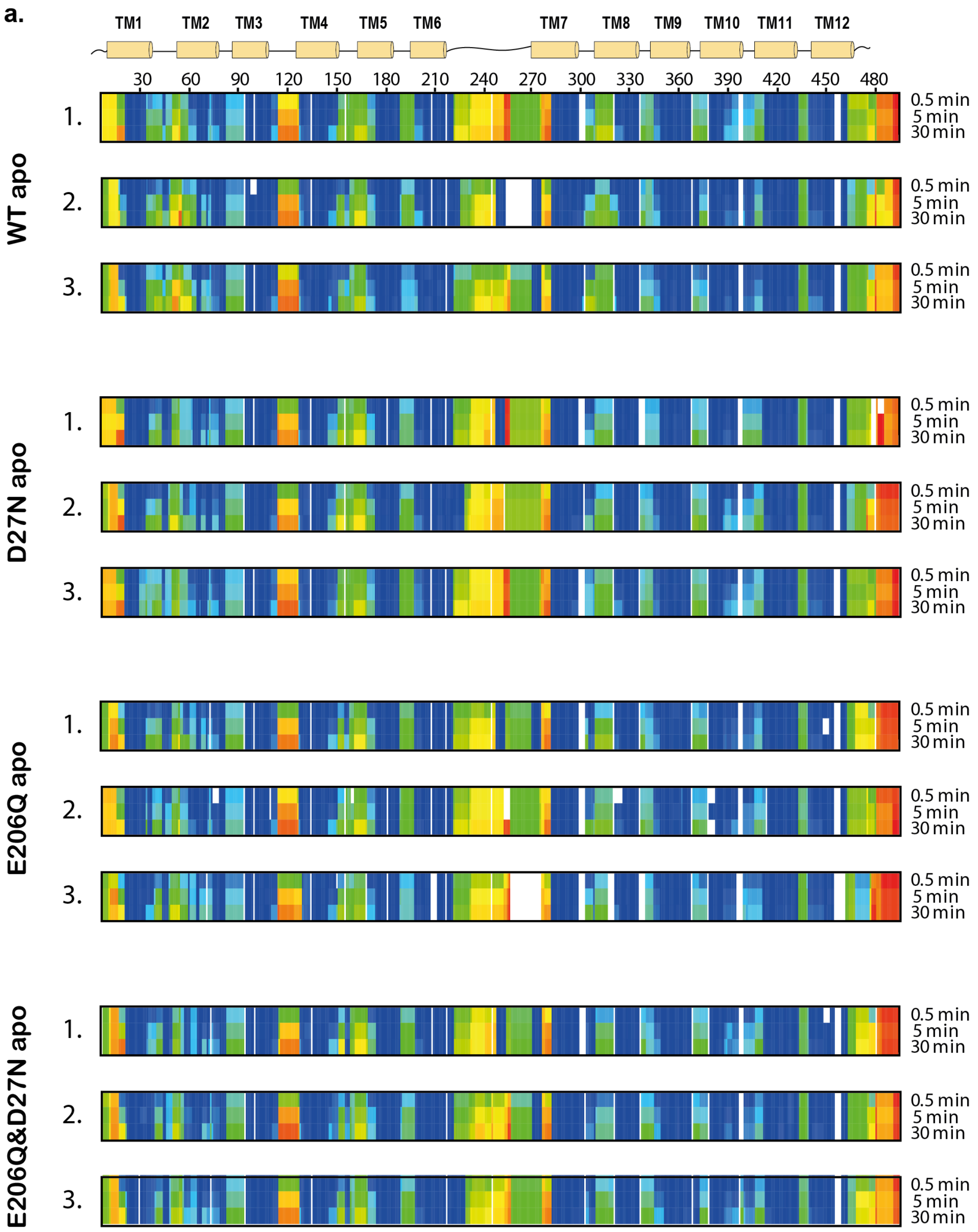

b.

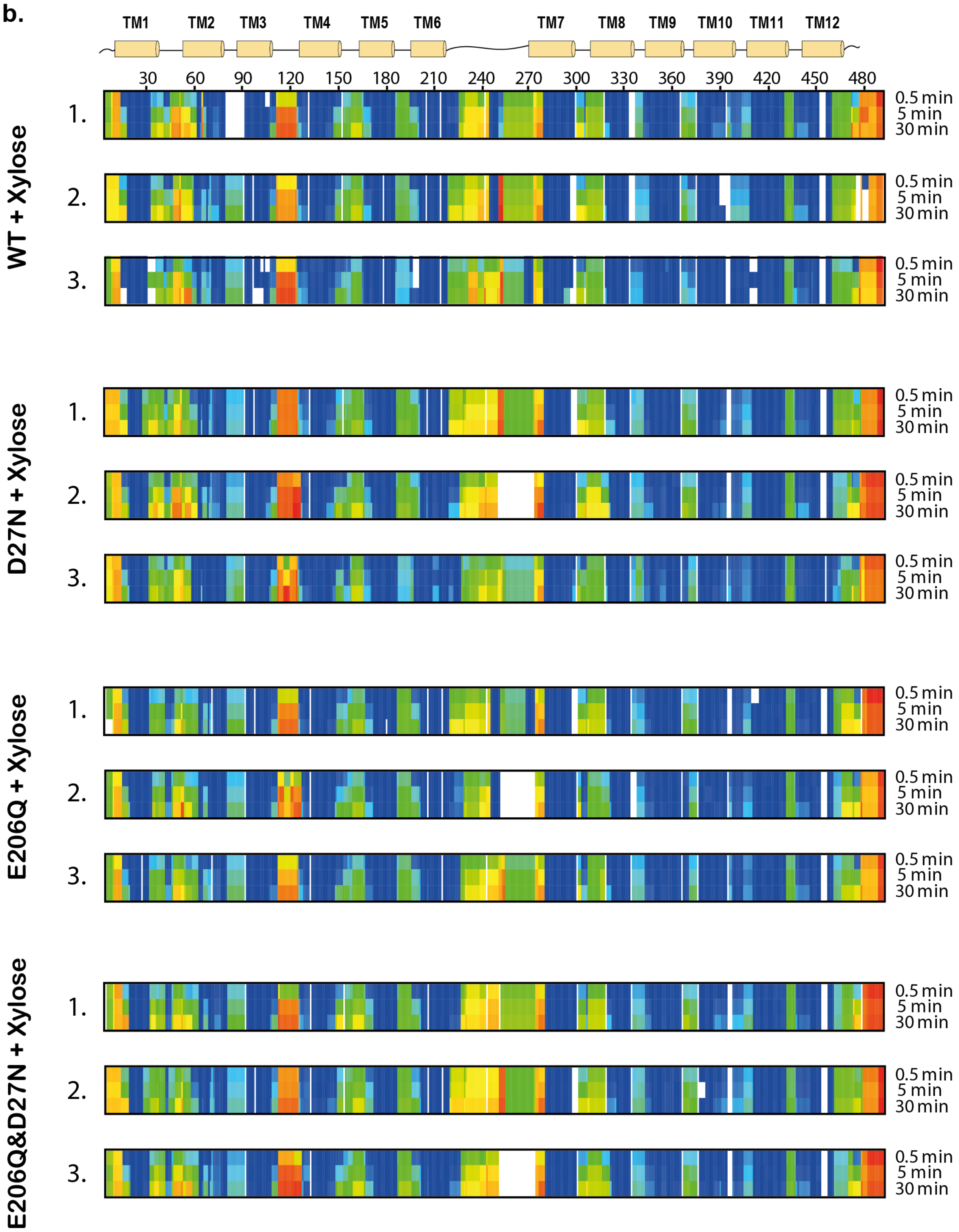
TM1 TM2 TM3 TM4 TM5 TM6 TM7 TM8 TM9 TM10 TM11 TM12
30 60 90 120 150 180 210 240 270 300 330 360 390 420 450 480
WT + Xylose
D27N + Xylose
E206Q + Xylose
E206Q&D27N + Xylose
1.
2.
3.
0.5 min
5 min
30 min

c.

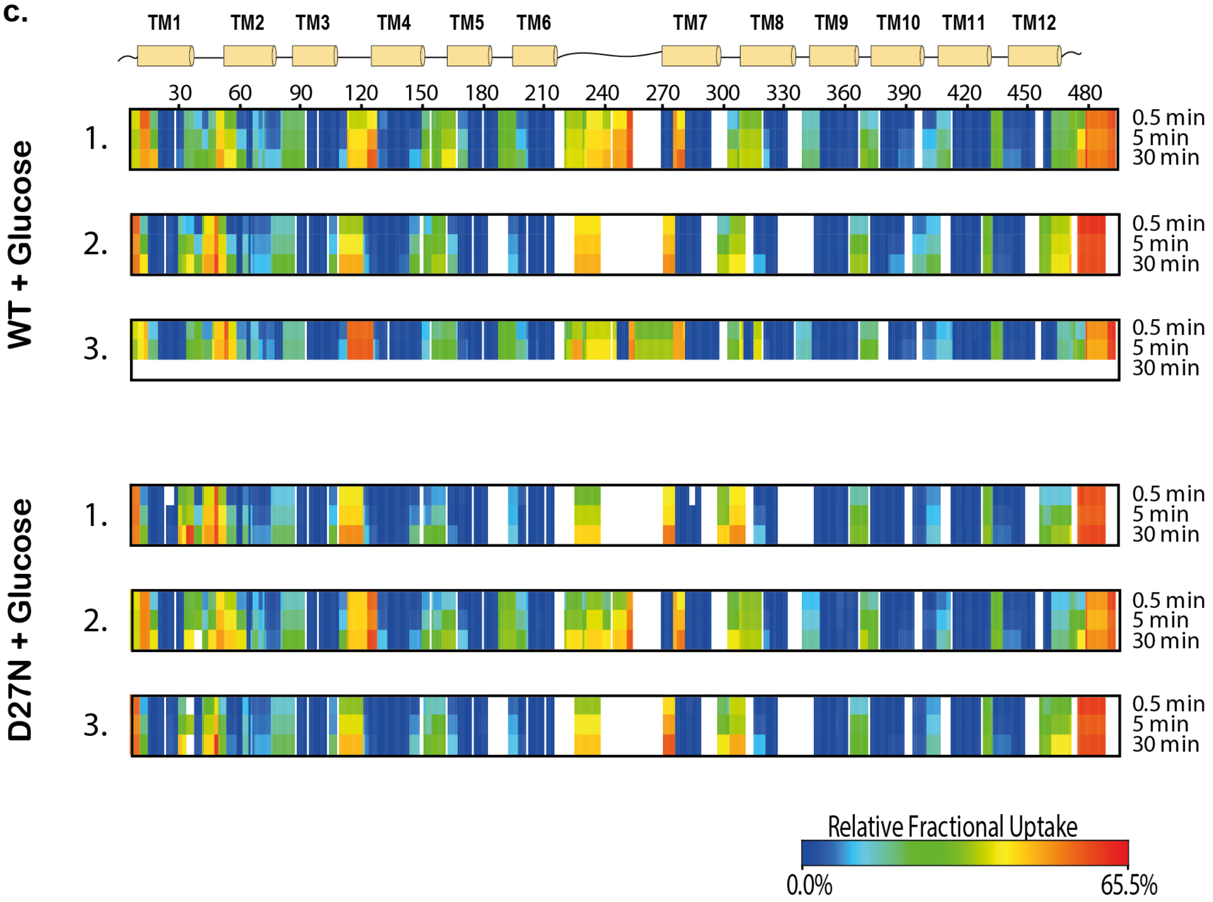

Supplementary Figure 2. Heat map of all protein states in triplicates in this study. (a). XylE WT, D27N, E206Q, and E206Q&D27N. **(c).** XylE WT, D27N, E206Q, and E206Q&D27N in the presence of substrate (xylose). **(c).** XylE WT and D27N in the presence of inhibitor (glucose).

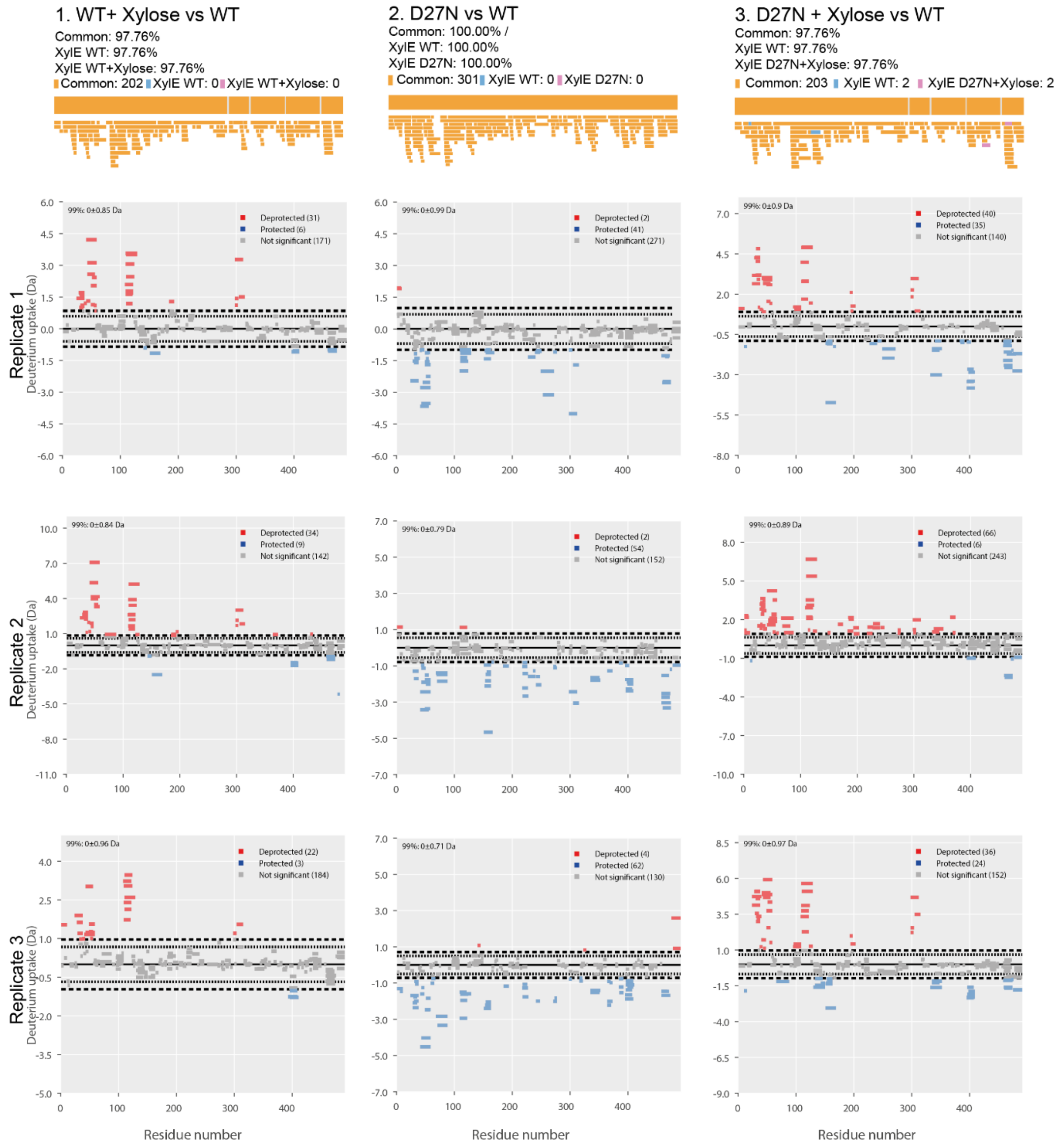
1. WT+ Xylose vs WT
Common: 97.76%
XylE WT: 97.76%
XylE WT+Xylose: 97.76%
Common: 202 XylE WT: 0 XylE WT+Xylose: 0
2. D27N vs WT
Common: 100.00% /
XylE WT: 100.00%
XylE D27N: 100.00%
Common: 301 XylE WT: 0 XylE D27N: 0
3. D27N + Xylose vs WT
Common: 97.76%
XylE WT: 97.76%
XylE D27N+Xylose: 97.76%
Common: 203 XylE WT: 2 XylE D27N+Xylose: 2
Replicate 1
Replicate 2
Replicate 3
Deuterium uptake (Da)
99%: 0±0.85 Da
Deprotected (31)
Protected (6)
Not significant (171)
99%: 0±0.99 Da
Deprotected (2)
Protected (41)
Not significant (271)
99%: 0±0.9 Da
Deprotected (40)
Protected (35)
Not significant (140)
99%: 0±0.84 Da
Deprotected (34)
Protected (9)
Not significant (142)
99%: 0±0.79 Da
Deprotected (2)
Protected (54)
Not significant (152)
99%: 0±0.89 Da
Deprotected (66)
Protected (6)
Not significant (243)
99%: 0±0.96 Da
Deprotected (22)
Protected (3)
Not significant (184)
99%: 0±0.71 Da
Deprotected (4)
Protected (62)
Not significant (130)
99%: 0±0.97 Da
Deprotected (36)
Protected (24)
Not significant (152)
Residue number

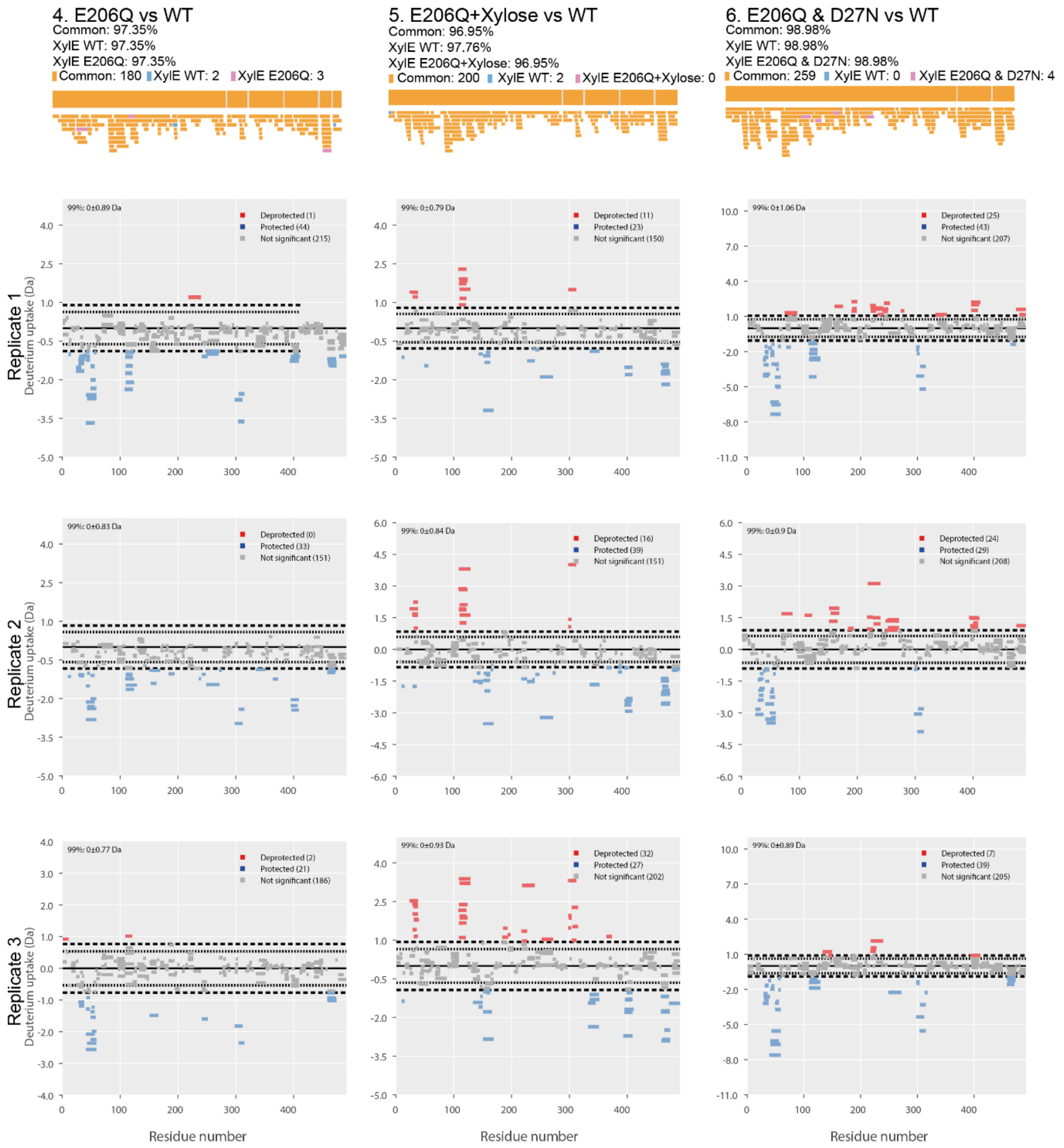
4. E206Q vs WT
Common: 97.35%
XylE WT: 97.35%
XylE E206Q: 97.35%
Common: 180 XylE WT: 2 XylE E206Q: 3
5. E206Q+Xylose vs WT
Common: 96.95%
XylE WT: 97.76%
XylE E206Q+Xylose: 96.95%
Common: 200 XylE WT: 2 XylE E206Q+Xylose: 0
6. E206Q & D27N vs WT
Common: 98.98%
XylE WT: 98.98%
XylE E206Q & D27N: 98.98%
Common: 259 XylE WT: 0 XylE E206Q & D27N: 4
Replicate 1
Replicate 2
Replicate 3
Deuterium uptake (Da)
99%: 0±0.89 Da
Deprotected (1)
Protected (44)
Not significant (215)
99%: 0±0.79 Da
Deprotected (11)
Protected (23)
Not significant (150)
99%: 0±1.06 Da
Deprotected (25)
Protected (43)
Not significant (207)
99%: 0±0.83 Da
Deprotected (0)
Protected (33)
Not significant (151)
99%: 0±0.84 Da
Deprotected (16)
Protected (39)
Not significant (151)
99%: 0±0.9 Da
Deprotected (24)
Protected (29)
Not significant (208)
99%: 0±0.77 Da
Deprotected (2)
Protected (21)
Not significant (186)
99%: 0±0.93 Da
Deprotected (32)
Protected (27)
Not significant (202)
99%: 0±0.89 Da
Deprotected (7)
Protected (39)
Not significant (205)
Residue number
Residue number
Residue number

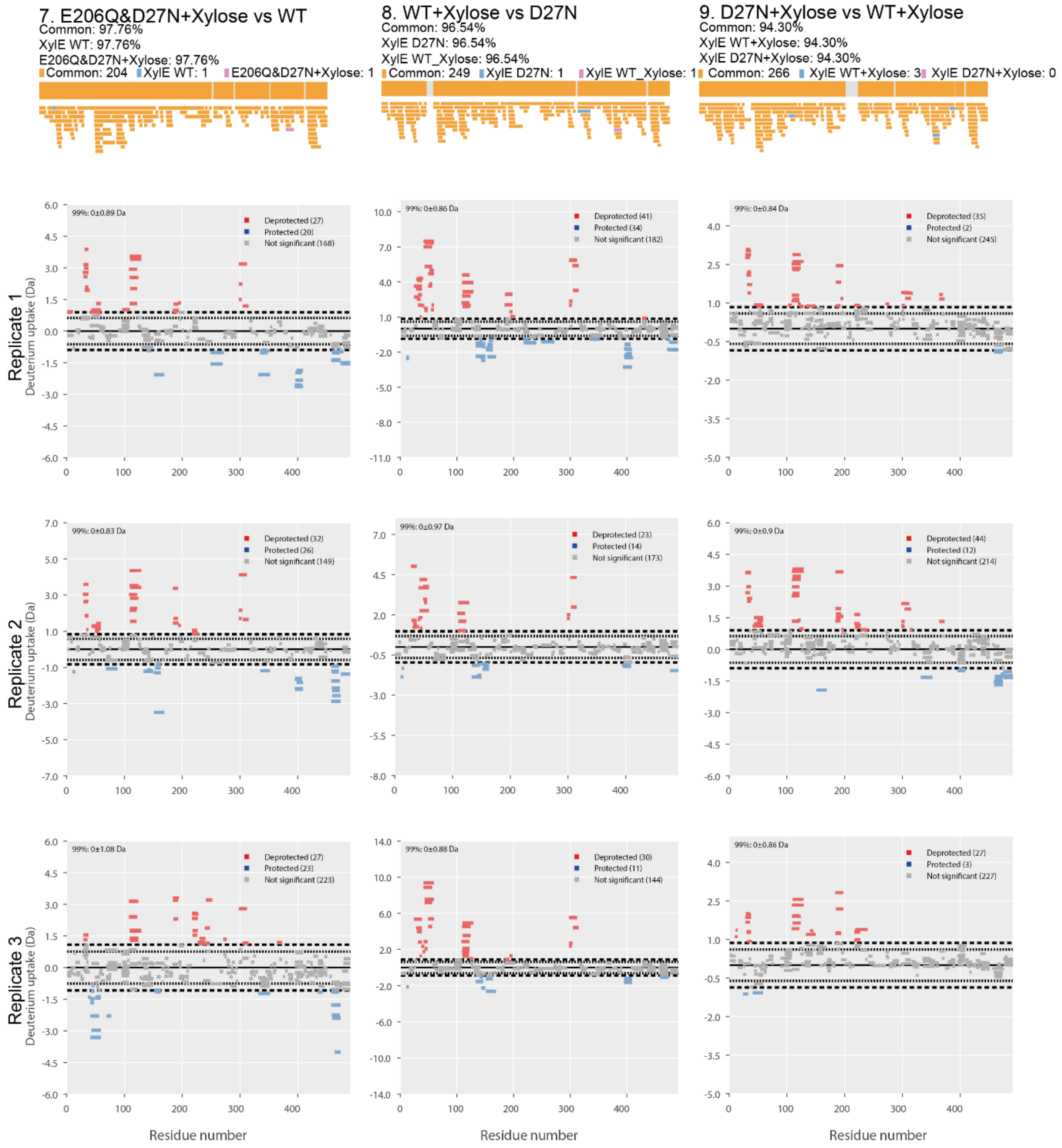

7. E206Q&D27N+Xylose vs WT
Common: 97.76%
XylE WT: 97.76%
E206Q&D27N+Xylose: 97.76%
Common: 204 XylE WT: 1 E206Q&D27N+Xylose: 1
8. WT+Xylose vs D27N
Common: 96.54%
XylE D27N: 96.54%
XylE WT_Xylose: 96.54%
Common: 249 XylE D27N: 1 XylE WT_Xylose: 1
9. D27N+Xylose vs WT+Xylose
Common: 94.30%
XylE WT+Xylose: 94.30%
XylE D27N+Xylose: 94.30%
Common: 266 XylE WT+Xylose: 3 XylE D27N+Xylose: 0
Replicate 1
Replicate 2
Replicate 3
Deuterium uptake (Da)
Residue number

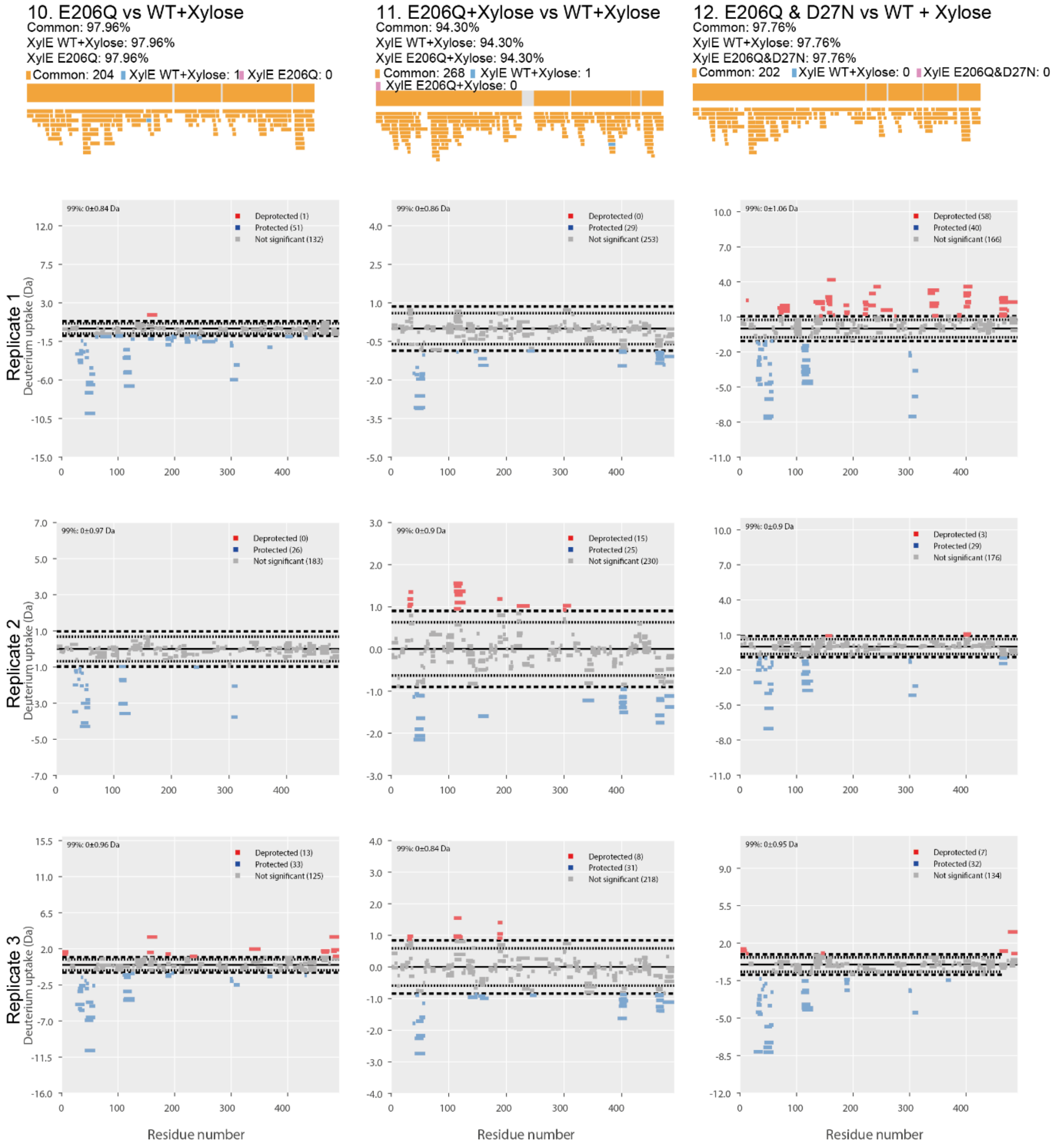
10. E206Q vs WT+Xylose
Common: 97.96%
XylE WT+Xylose: 97.96%
XylE E206Q: 97.96%
Common: 204 XylE WT+Xylose: 1 XylE E206Q: 0
11. E206Q+Xylose vs WT+Xylose
Common: 94.30%
XylE WT+Xylose: 94.30%
XylE E206Q+Xylose: 94.30%
Common: 268 XylE WT+Xylose: 1
XylE E206Q+Xylose: 0
12. E206Q & D27N vs WT + Xylose
Common: 97.76%
XylE WT+Xylose: 97.76%
XylE E206Q&D27N: 97.76%
Common: 202 XylE WT+Xylose: 0 XylE E206Q&D27N: 0
Replicate 1
Replicate 2
Replicate 3
Deuterium uptake (Da)
99%: 0±0.84 Da
Deprotected (1)
Protected (51)
Not significant (132)
99%: 0±0.86 Da
Deprotected (0)
Protected (29)
Not significant (253)
99%: 0±1.06 Da
Deprotected (58)
Protected (40)
Not significant (166)
99%: 0±0.97 Da
Deprotected (0)
Protected (26)
Not significant (183)
99%: 0±0.9 Da
Deprotected (15)
Protected (25)
Not significant (230)
99%: 0±0.9 Da
Deprotected (3)
Protected (29)
Not significant (176)
99%: 0±0.96 Da
Deprotected (13)
Protected (33)
Not significant (125)
99%: 0±0.84 Da
Deprotected (8)
Protected (31)
Not significant (218)
99%: 0±0.95 Da
Deprotected (7)
Protected (32)
Not significant (134)
Residue number
Residue number
Residue number

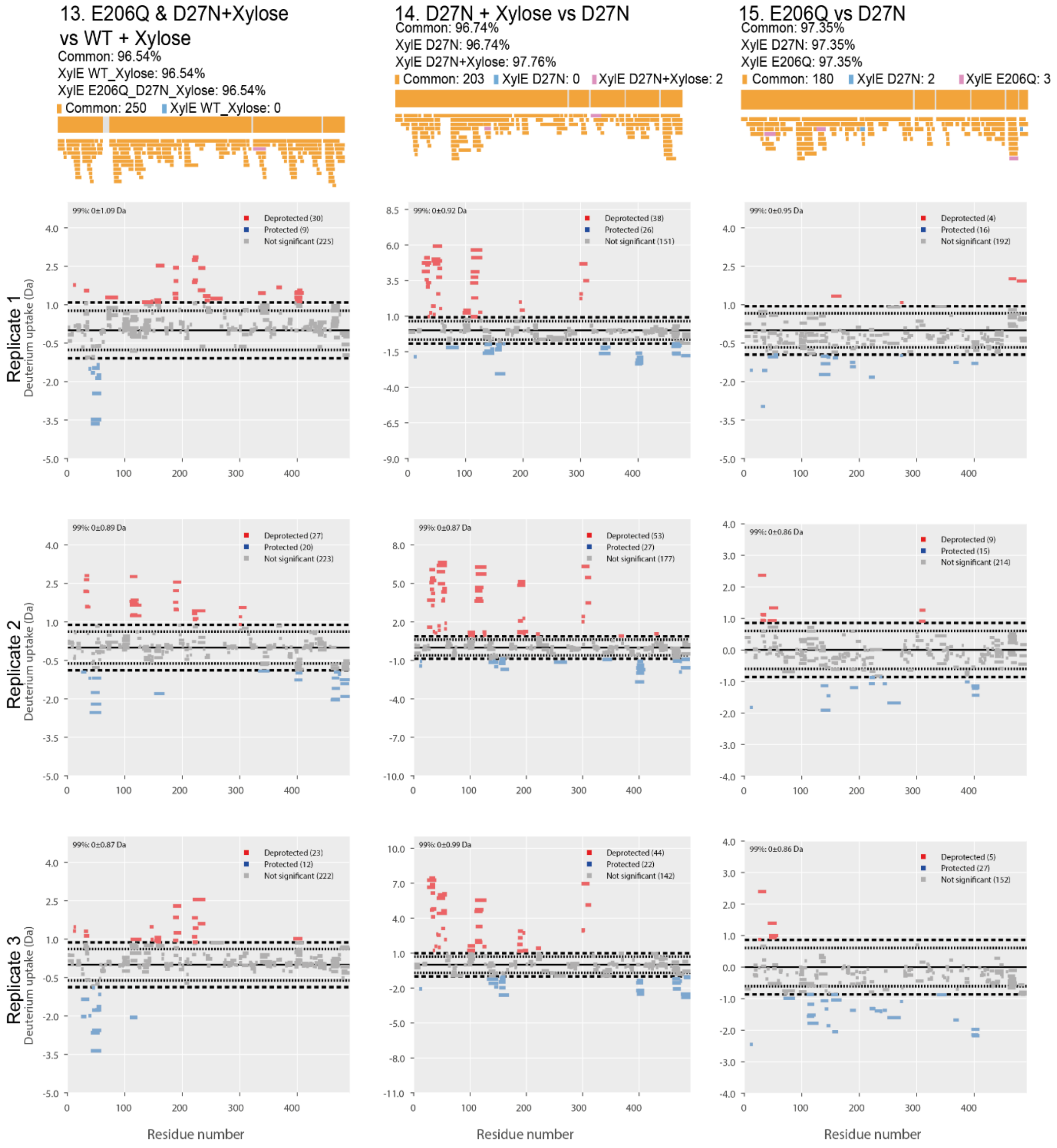
13. E206Q & D27N+Xylose vs WT + Xylose
Common: 96.54%
XylE WT_Xylose: 96.54%
XylE E206Q_D27N_Xylose: 96.54%
Common: 250
XylE WT_Xylose: 0
14. D27N + Xylose vs D27N
Common: 96.74%
XylE D27N: 96.74%
XylE D27N+Xylose: 97.76%
Common: 203
XylE D27N: 0
XylE D27N+Xylose: 2
15. E206Q vs D27N
Common: 97.35%
XylE D27N: 97.35%
XylE E206Q: 97.35%
Common: 180
XylE D27N: 2
XylE E206Q: 3
Replicate 1
Replicate 2
Replicate 3
Deuterium uptake (Da)
Residue number

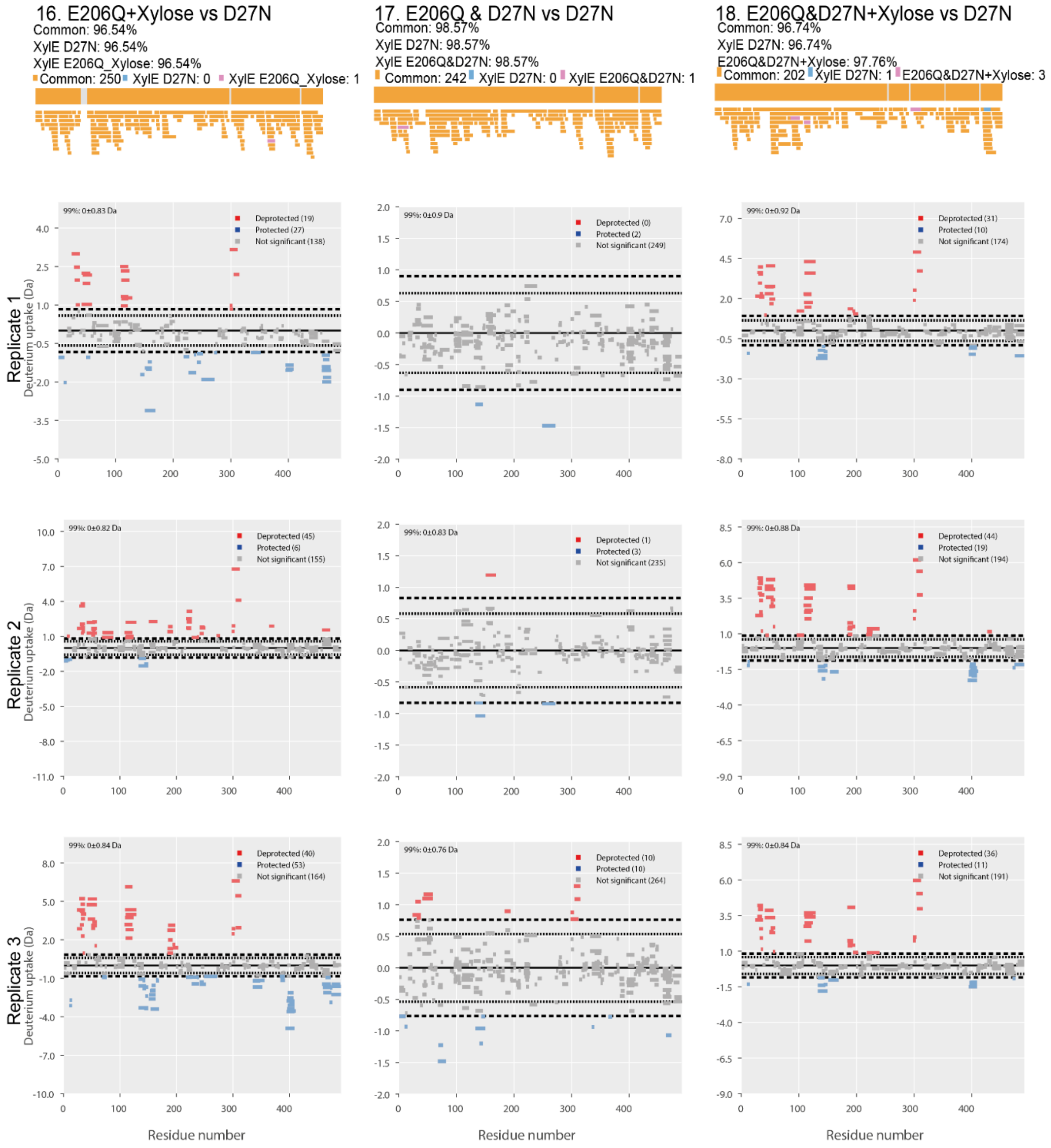
16. E206Q+Xylose vs D27N
Common: 96.54%
XylE D27N: 96.54%
XylE E206Q_Xylose: 96.54%
Common: 250 XylE D27N: 0 XylE E206Q_Xylose: 1
17. E206Q & D27N vs D27N
Common: 98.57%
XylE D27N: 98.57%
XylE E206Q&D27N: 98.57%
Common: 242 XylE D27N: 0 XylE E206Q&D27N: 1
18. E206Q&D27N+Xylose vs D27N
Common: 96.74%
XylE D27N: 96.74%
E206Q&D27N+Xylose: 97.76%
Common: 202 XylE D27N: 1 E206Q&D27N+Xylose: 3
Replicate 1
Replicate 2
Replicate 3
Deuterium uptake (Da)
99%: 0±0.83 Da
Deprotected (19)
Protected (27)
Not significant (138)
99%: 0±0.9 Da
Deprotected (0)
Protected (2)
Not significant (249)
99%: 0±0.92 Da
Deprotected (31)
Protected (10)
Not significant (174)
99%: 0±0.82 Da
Deprotected (45)
Protected (6)
Not significant (155)
99%: 0±0.83 Da
Deprotected (1)
Protected (3)
Not significant (235)
99%: 0±0.88 Da
Deprotected (44)
Protected (19)
Not significant (194)
99%: 0±0.84 Da
Deprotected (40)
Protected (53)
Not significant (164)
99%: 0±0.76 Da
Deprotected (10)
Protected (10)
Not significant (264)
99%: 0±0.84 Da
Deprotected (36)
Protected (11)
Not significant (191)
Residue number

19. D27N + Xylose vs E206Q

Common: 97.76%
XylE E206Q: 97.76%
XylE D27N+Xylose: 97.76%
Common: 202 XylE E206Q: 4 XylE D27N+Xylose: 3

20.D27N +Xylose vs E206Q +Xylose

Common: 93.89%
XylE D27N+Xylose: 93.89%
XylE E206Q+Xylose: 93.89%
Common: 247 XylE D27N+Xylose: 9
XylE E206Q+Xylose: 5

21. E206Q&D27N vs D27N+Xylose

Common: 98.98%
XylE D27N_Xylose: 98.98%
XylE E206Q_D27N: 98.98%
Common: 230 XylE D27N_Xylose: 1 XylE E206Q_D27N: 0

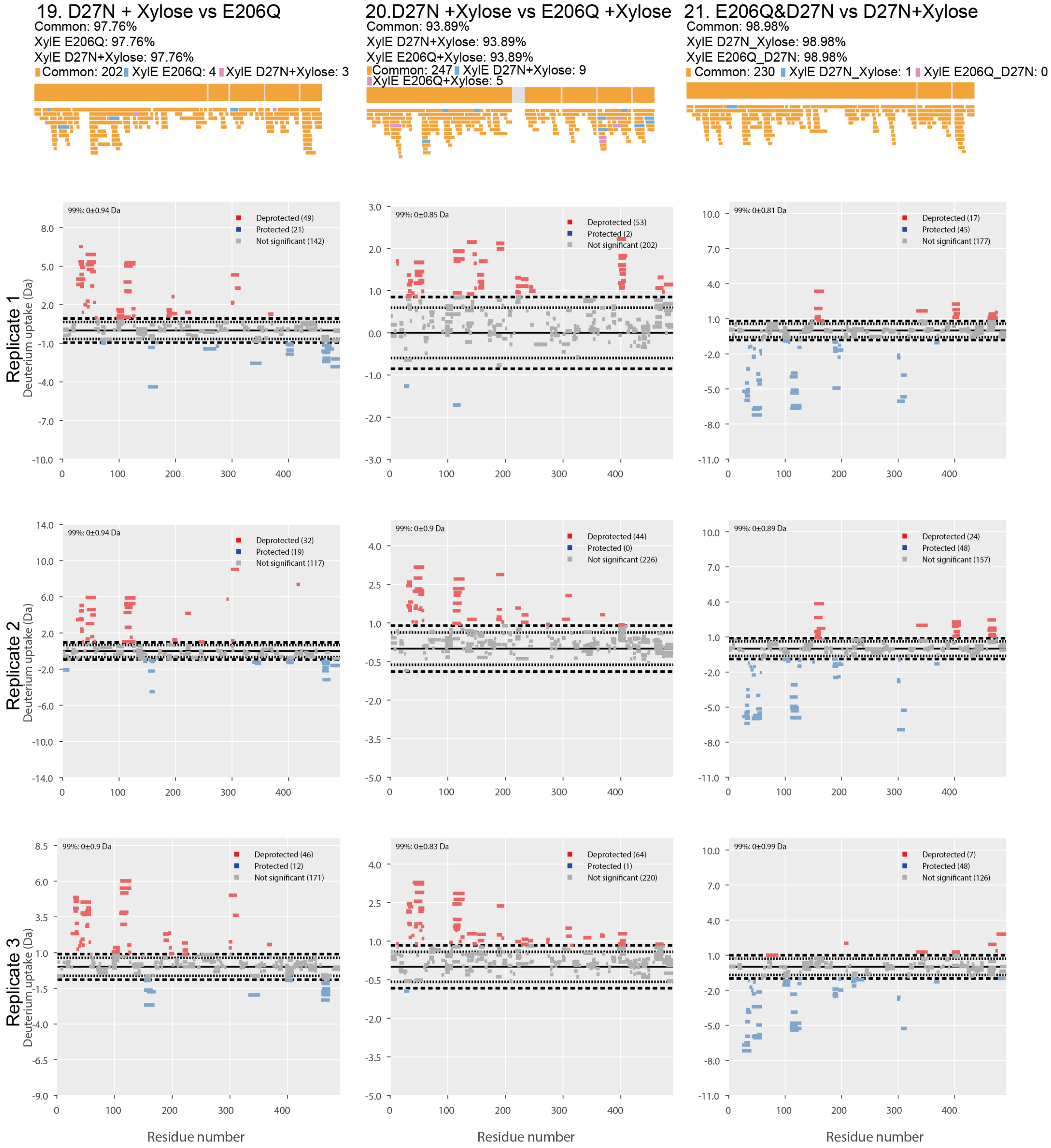

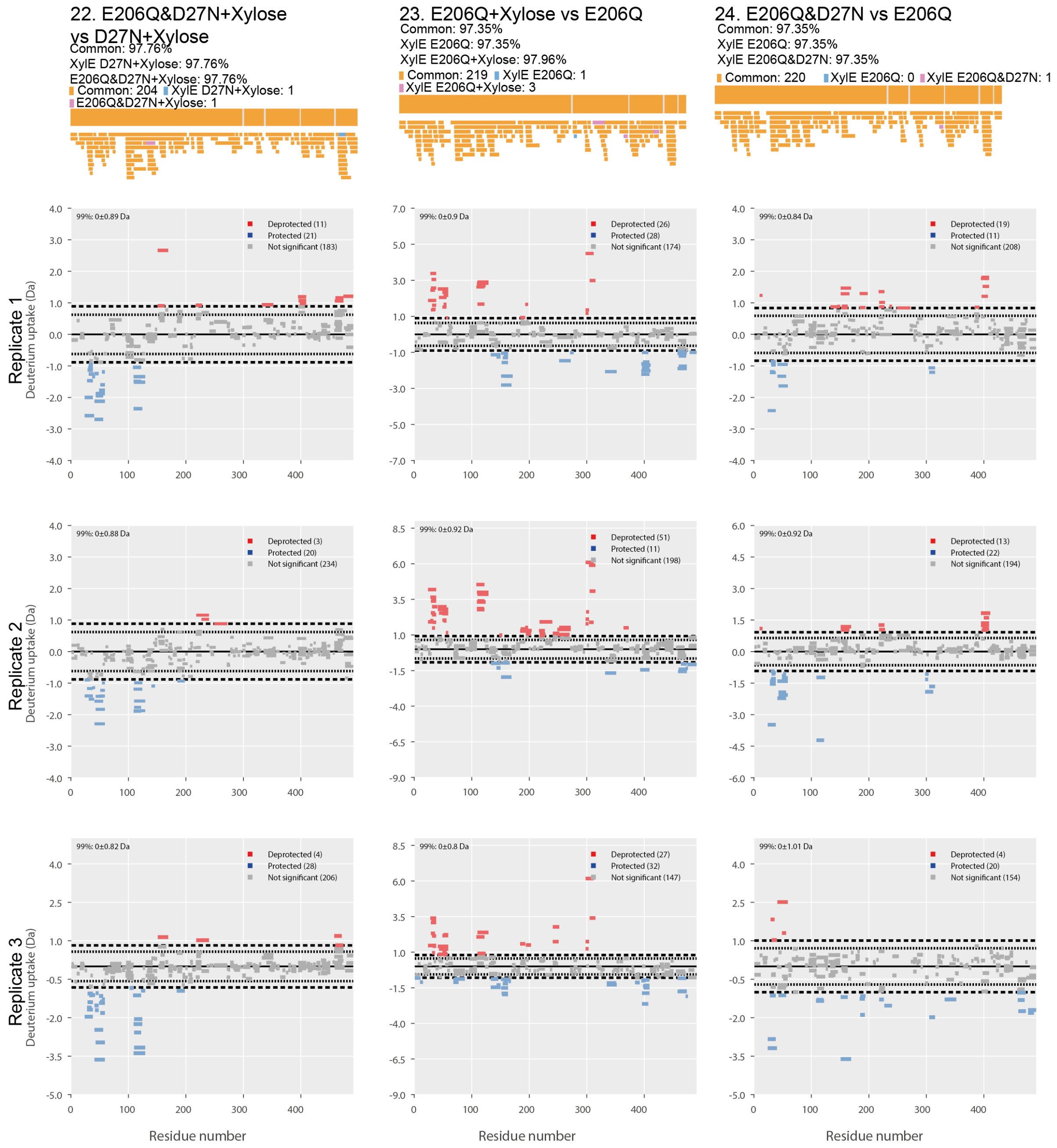
22. E206Q&D27N+Xylose vs D27N+Xylose
Common: 97.76%
XylE D27N+Xylose: 97.76%
E206Q&D27N+Xylose: 97.76%
Common: 204
XylE D27N+Xylose: 1
E206Q&D27N+Xylose: 1
23. E206Q+Xylose vs E206Q
Common: 97.35%
XylE E206Q: 97.35%
XylE E206Q+Xylose: 97.96%
Common: 219
XylE E206Q: 1
XylE E206Q+Xylose: 3
24. E206Q&D27N vs E206Q
Common: 97.35%
XylE E206Q: 97.35%
XylE E206Q&D27N: 97.35%
Common: 220
XylE E206Q: 0
XylE E206Q&D27N: 1
Replicate 1
Replicate 2
Replicate 3
Deuterium uptake (Da)
99%: 0±0.89 Da
Deprotected (11)
Protected (21)
Not significant (183)
99%: 0±0.9 Da
Deprotected (26)
Protected (28)
Not significant (174)
99%: 0±0.84 Da
Deprotected (19)
Protected (11)
Not significant (208)
99%: 0±0.88 Da
Deprotected (3)
Protected (20)
Not significant (234)
99%: 0±0.92 Da
Deprotected (51)
Protected (11)
Not significant (198)
99%: 0±0.92 Da
Deprotected (13)
Protected (22)
Not significant (194)
99%: 0±0.82 Da
Deprotected (4)
Protected (28)
Not significant (206)
99%: 0±0.8 Da
Deprotected (27)
Protected (32)
Not significant (147)
99%: 0±1.01 Da
Deprotected (4)
Protected (20)
Not significant (154)
Residue number

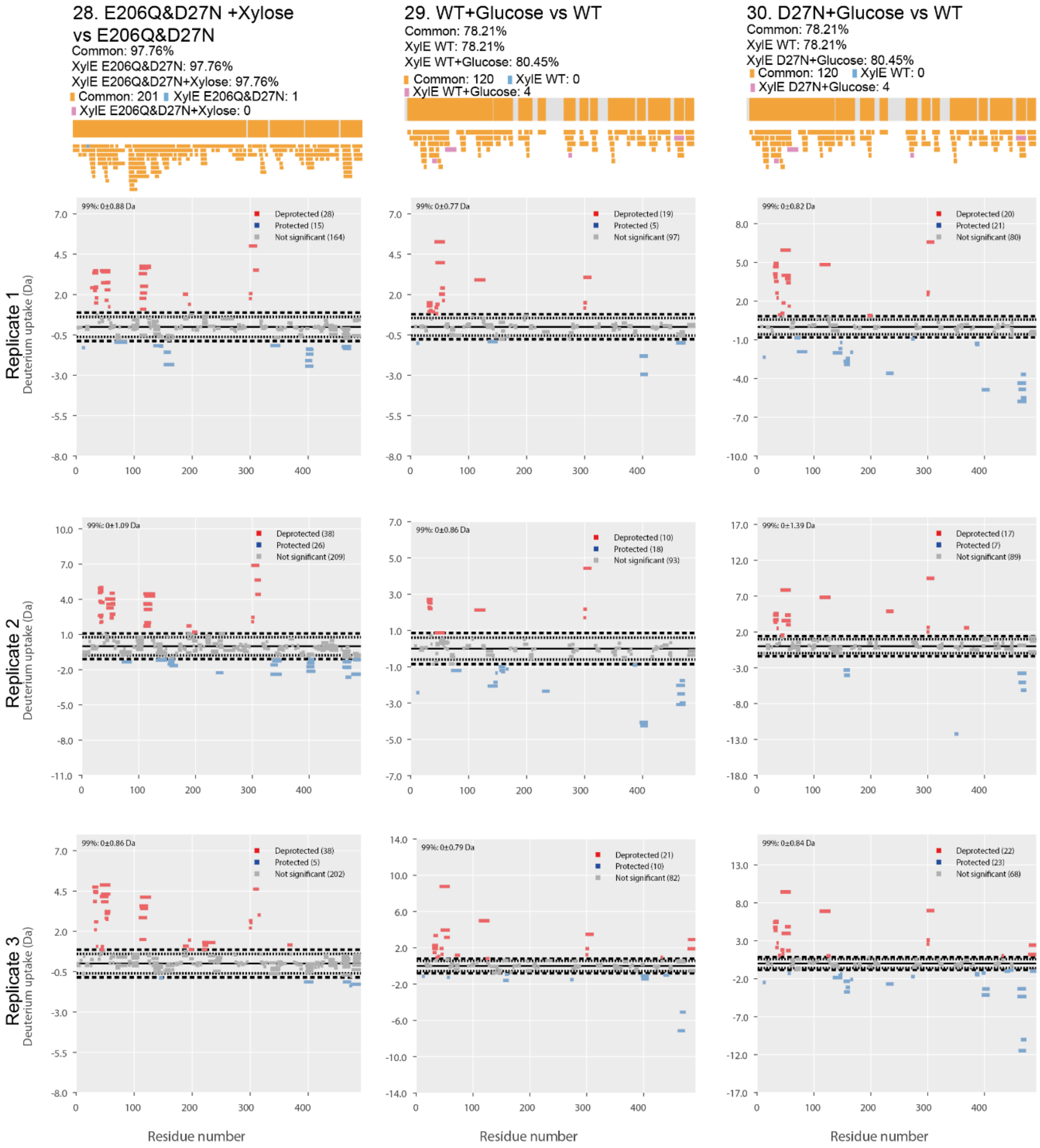
28. E206Q&D27N +Xylose vs E206Q&D27N
Common: 97.76%
XylE E206Q&D27N: 97.76%
XylE E206Q&D27N+Xylose: 97.76%
Common: 201 XylE E206Q&D27N: 1
XylE E206Q&D27N+Xylose: 0
29. WT+Glucose vs WT
Common: 78.21%
XylE WT: 78.21%
XylE WT+Glucose: 80.45%
Common: 120 XylE WT: 0
XylE WT+Glucose: 4
30. D27N+Glucose vs WT
Common: 78.21%
XylE WT: 78.21%
XylE D27N+Glucose: 80.45%
Common: 120 XylE WT: 0
XylE D27N+Glucose: 4
Replicate 1
Replicate 2
Replicate 3
Deuterium uptake (Da)
99%: 0±0.88 Da
Deprotected (28)
Protected (15)
Not significant (164)
99%: 0±0.77 Da
Deprotected (19)
Protected (5)
Not significant (97)
99%: 0±0.82 Da
Deprotected (20)
Protected (21)
Not significant (80)
99%: 0±1.09 Da
Deprotected (38)
Protected (26)
Not significant (209)
99%: 0±0.86 Da
Deprotected (10)
Protected (18)
Not significant (93)
99%: 0±1.39 Da
Deprotected (17)
Protected (7)
Not significant (89)
99%: 0±0.86 Da
Deprotected (38)
Protected (5)
Not significant (202)
99%: 0±0.79 Da
Deprotected (21)
Protected (10)
Not significant (82)
99%: 0±0.84 Da
Deprotected (22)
Protected (23)
Not significant (68)
Residue number

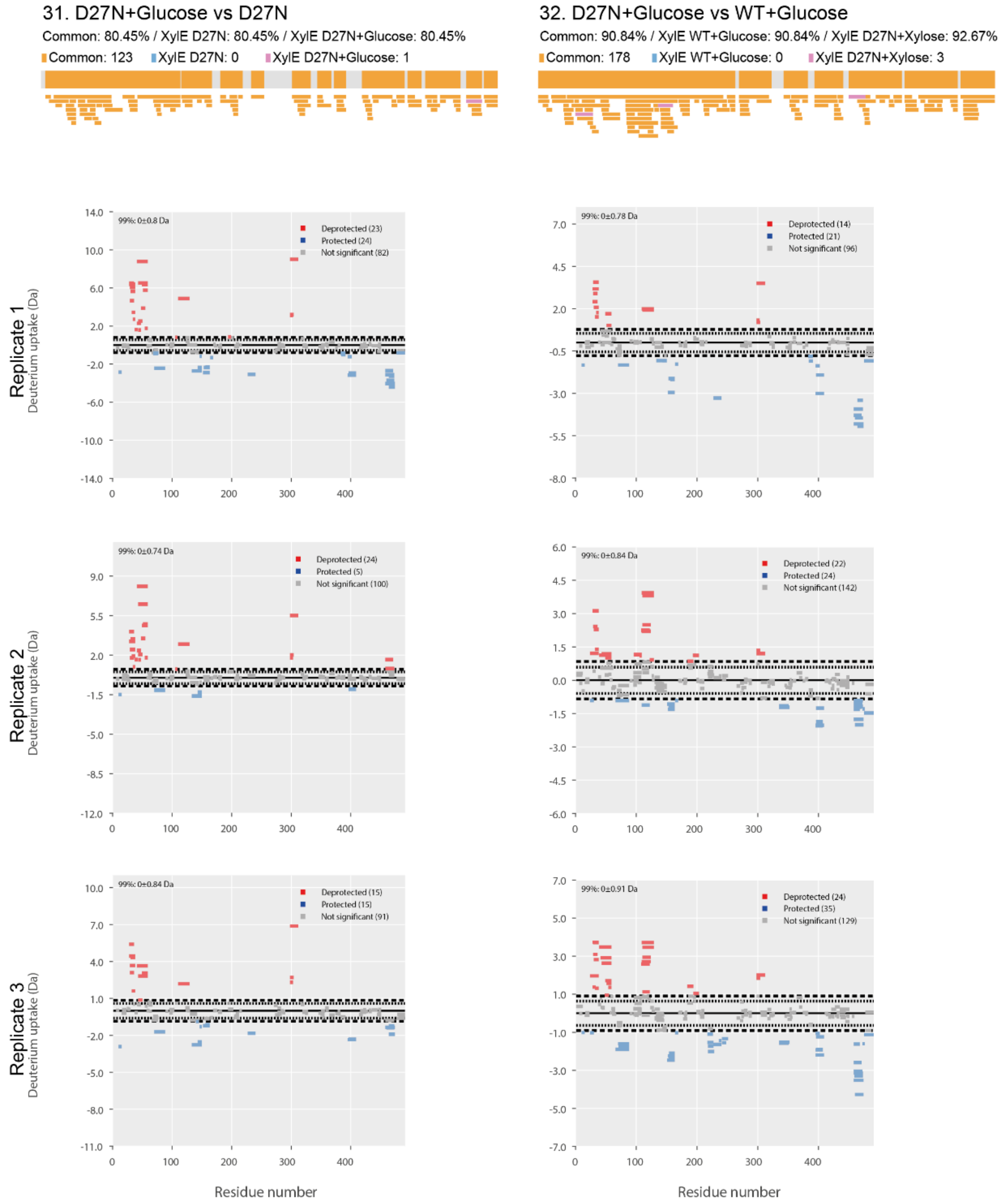

Supplementary Figure 3. Woods plots (in triplicates) and comparative sequence coverage maps obtained from differential HDX of XylE. Each bar represents a single peptide with peptide length indicated by the bar length. Common peptide between two different protein states are indicated as orange, unique peptides are in blue and pink separately.

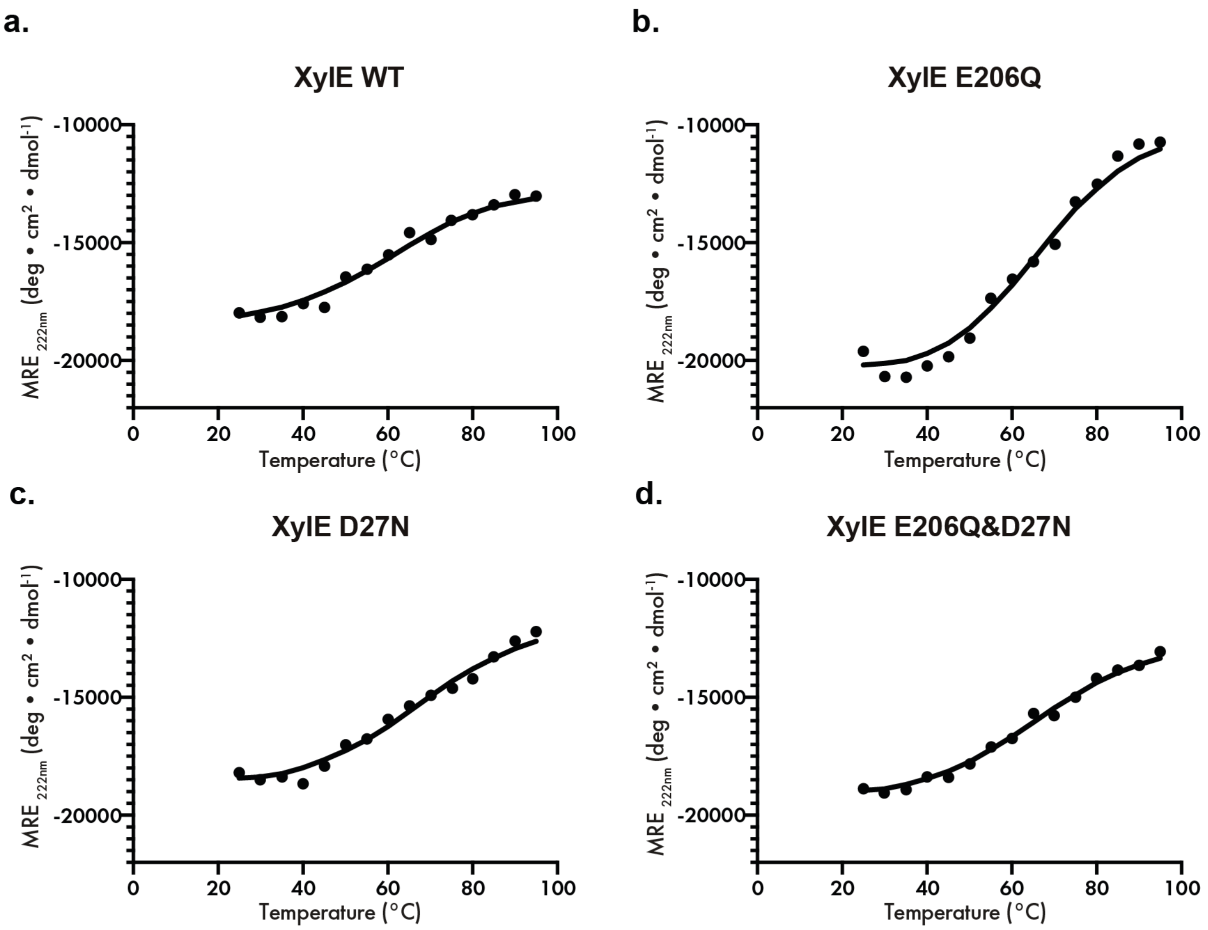

Supplementary Figure 4. Thermal denaturation Circular Dichroism of XylE wild type, E206Q, D27N and E206Q&D27N. (**a-d). ** Measurements of XylE WT, E206Q, D27N, and E206Q&D27N changes in MRE (Mean Residue Ellipticity) at fixed wavelength 222 nm from 25 ℃ to 95 ℃.

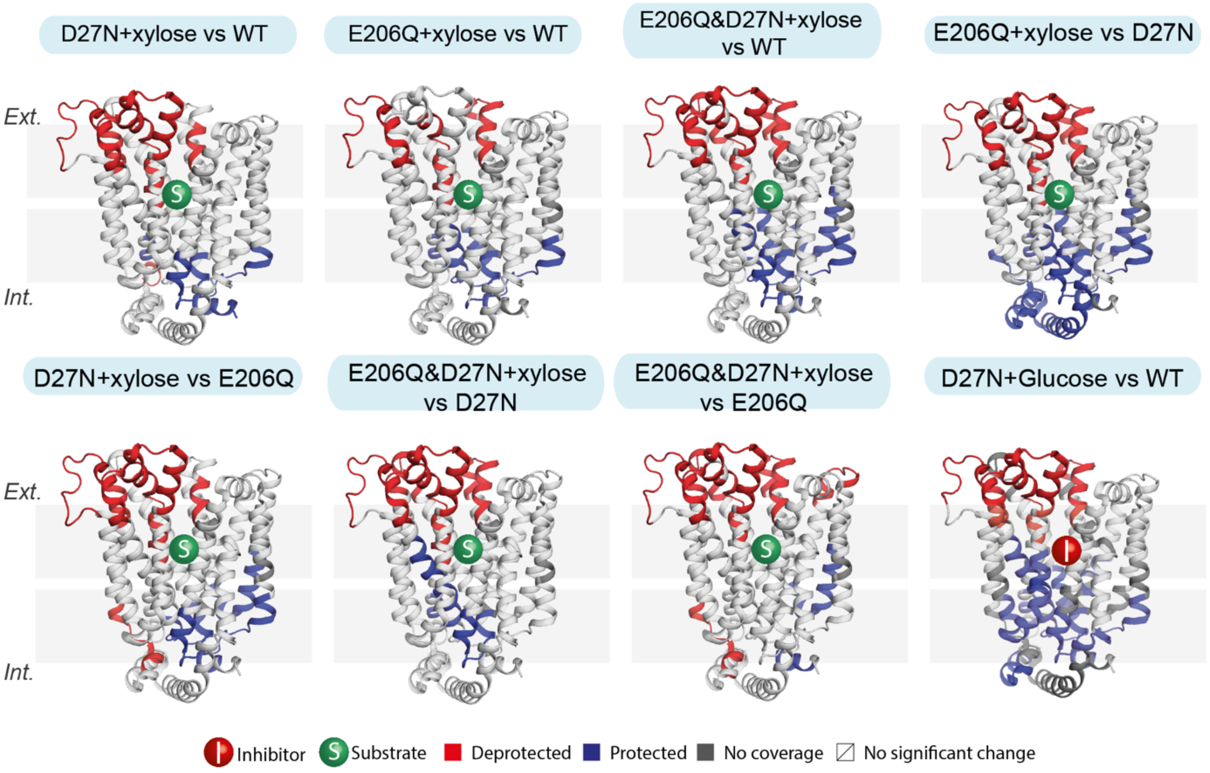

Supplementary Figure 5. Conformational equilibrium towards outward-facing upon ligand (xylose and glucose) binding. Figures are made with Pymol using the 3D protein structure (PDB: 4GBY). Blue and red regions indicate a relatively negative (protected) and a positive (deprotected) deuterium uptake differences respectively.

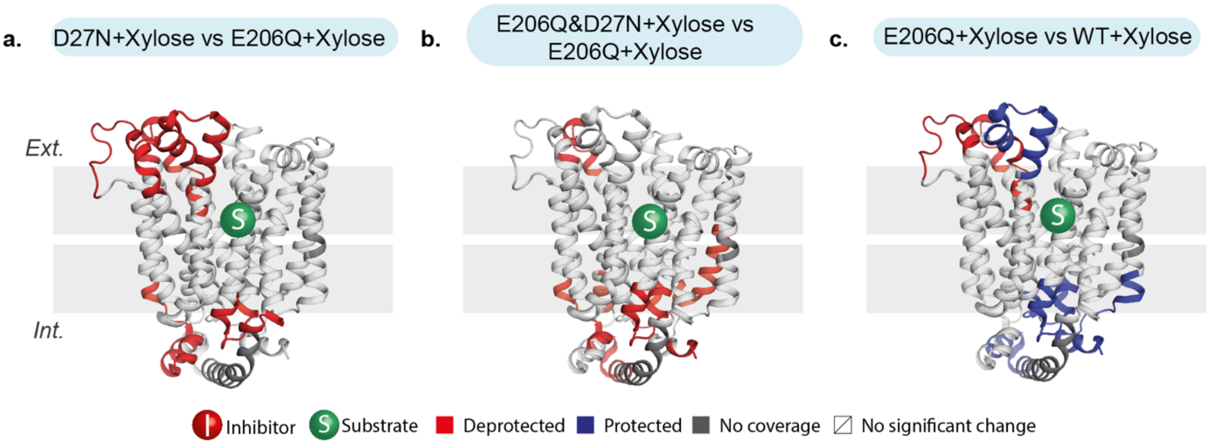

Supplementary Figure 6. Differential deuterium uptake between XylE (a) D27N and E206Q, (b) E206Q&D27N and E206Q, and (c) E206Q and WT. All measurements were performed in the presence of substrate (xylose).

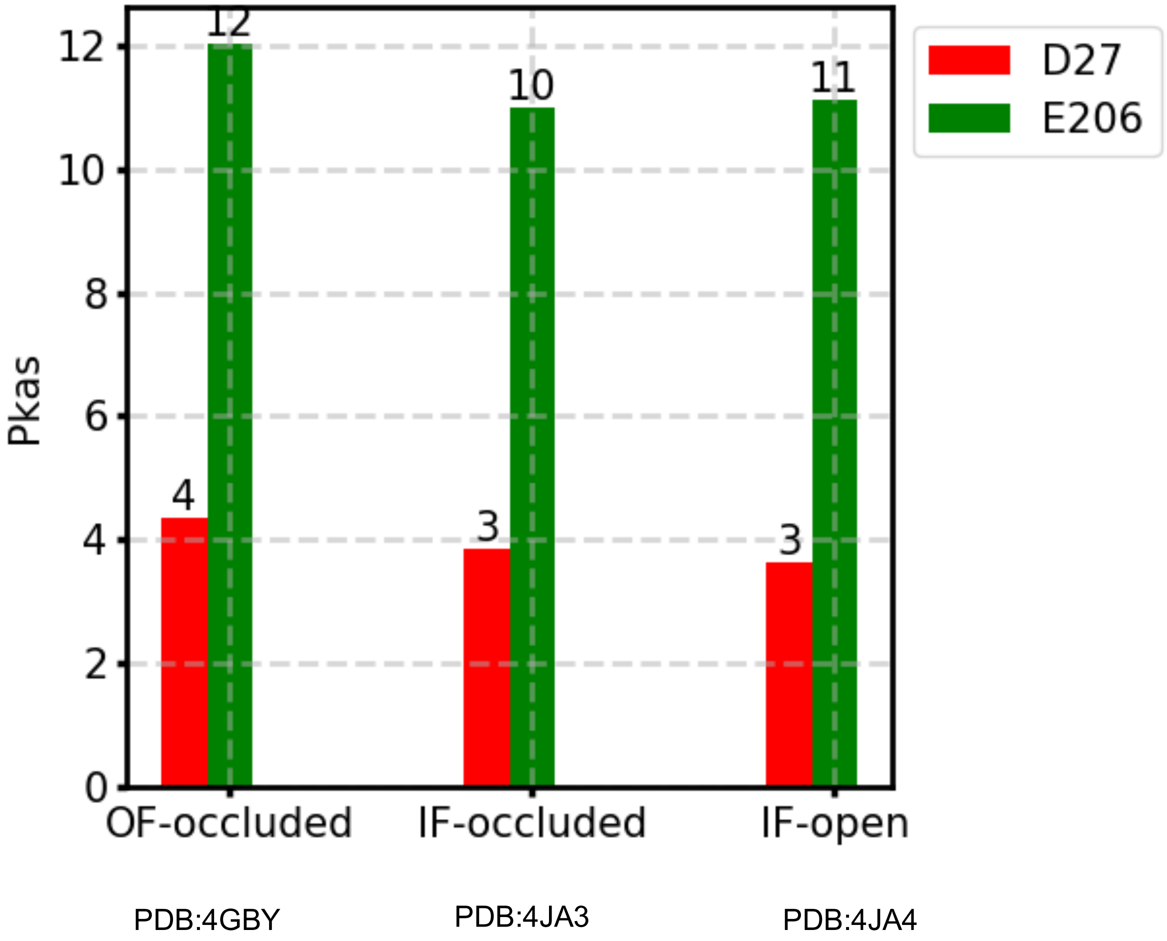

Supplementary Figure 7. pKa values predicted by PROPKA server [2] for three conformational states of XylE.

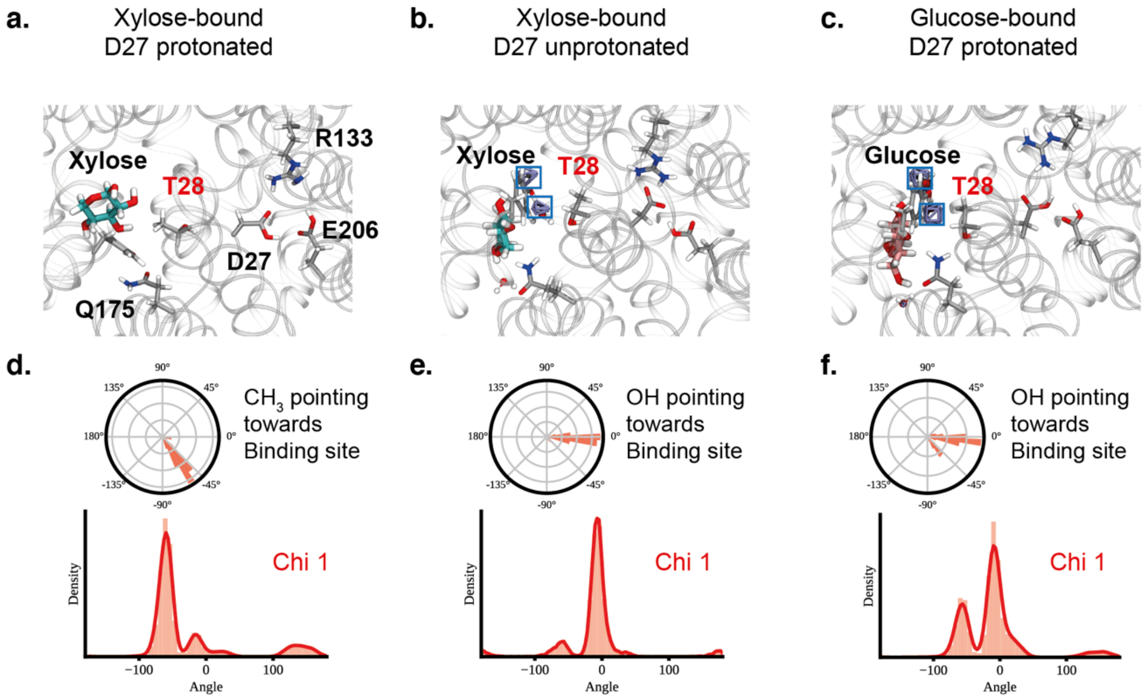

Supplementary Figure 8. Coupling between TM1 and the XylE substrate binding site. Snapshots of simulations show that T28 has a different orientation in **(a)** the xylose-bound / protonated D27 structure compared to xylose-bound unprotonated D27 and glucose-bound protonated D27. Ordered water molecules (blue) are interacting with T28 in **(b)** and **(c)**. Dihedral angles of T28 shows a different orientation for **(d)** xylose-bound protonated case compared to **(e-f)**.

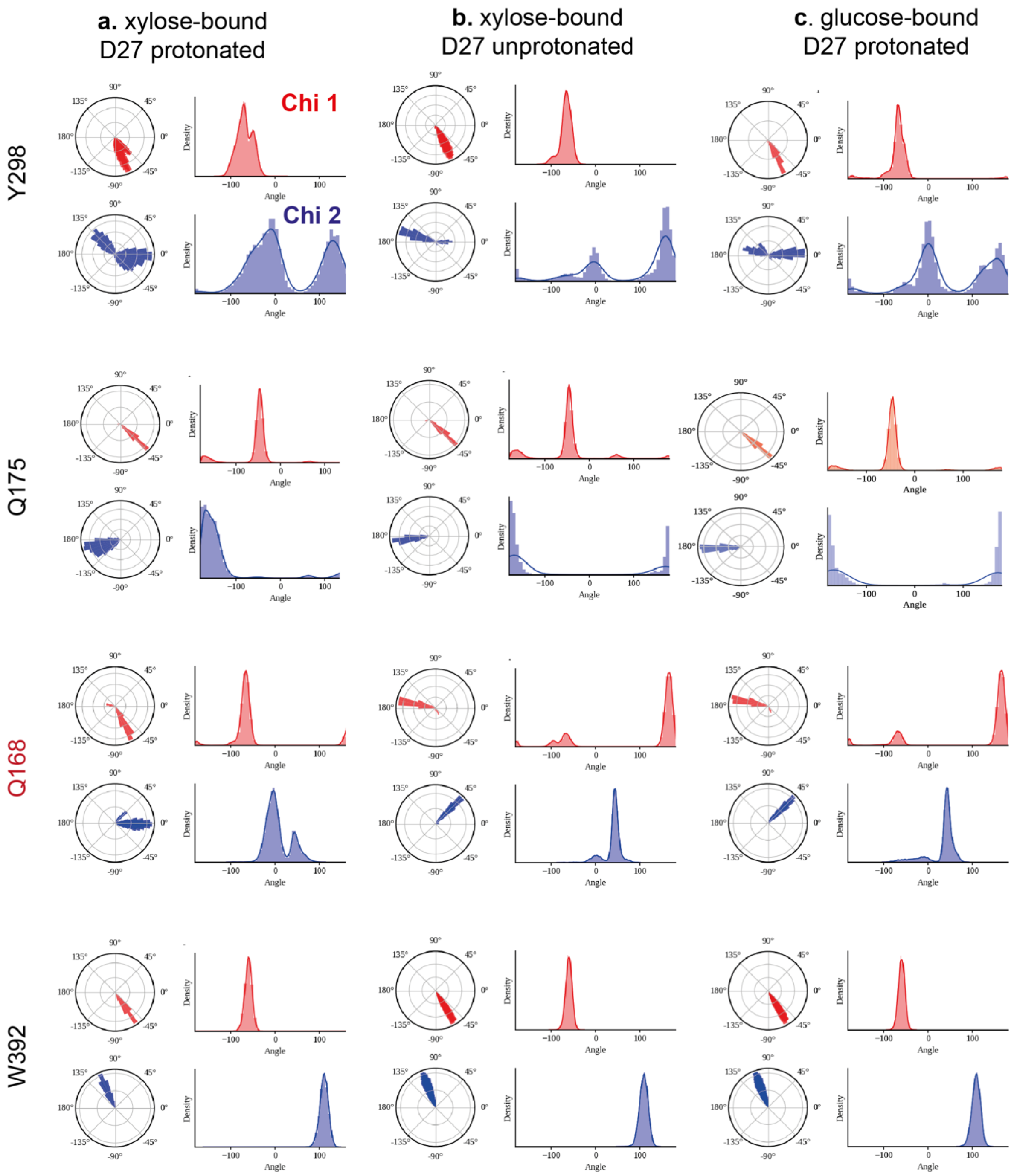

a. xylose-bound D27 protonated
b. xylose-bound D27 unprotonated
c. glucose-bound D27 protonated
Y298
Q175
Q168
W392
Chi 1
Chi 2
Density
Angle

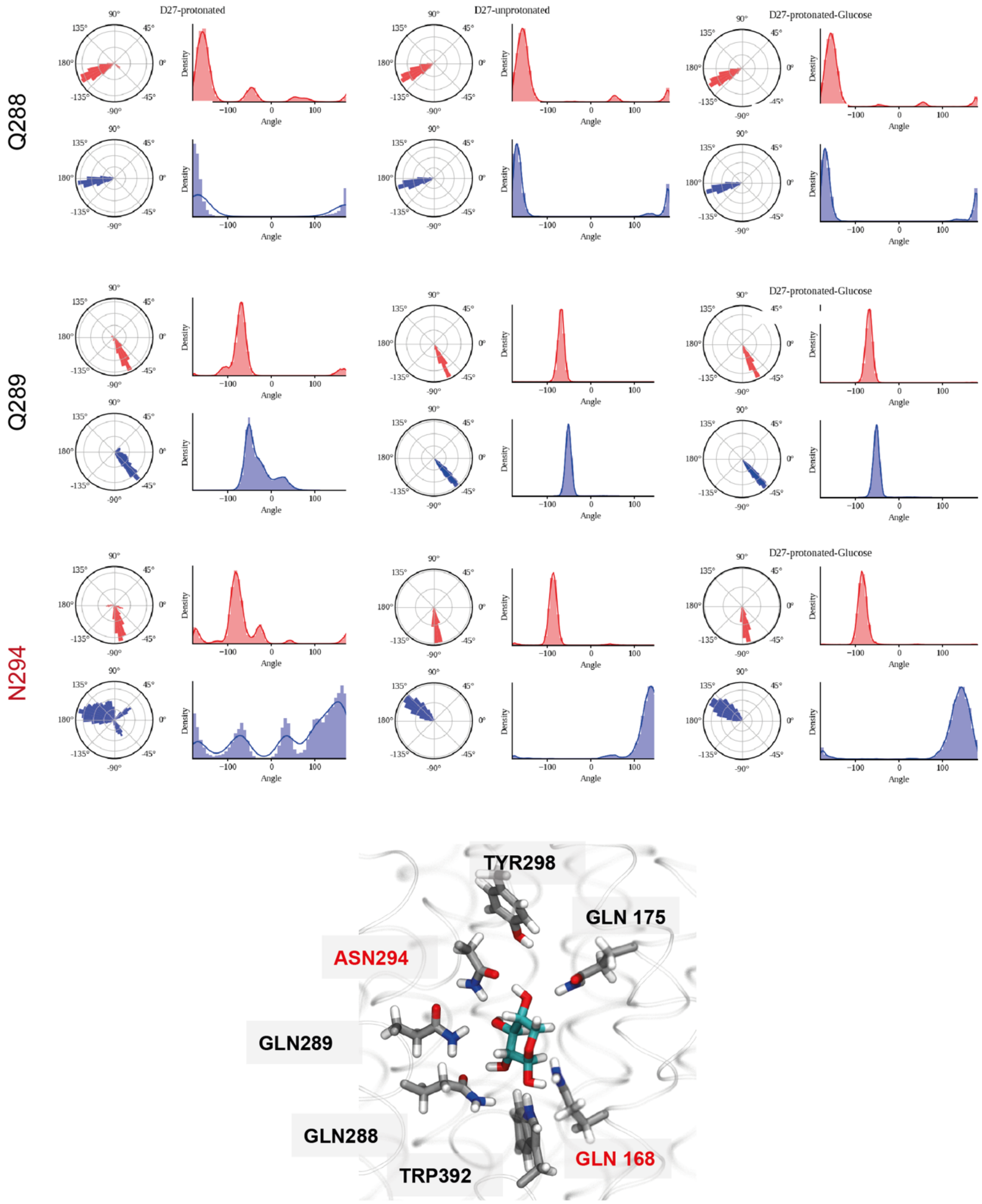

Supplementary Figure 9. Dihedral angles of seven XylE residues involved in substrate binding. Plots depict the orientation of each residue side chain across three states of XylE: **(a)** xylose-bound D27 protonated, **(b)** xylose-bound D27 unprotonated and **(c)** glucose-bound D27 protonated. Residues showing a different orientation in the xylose-bound D27 protonated case are highlighted in red (Q168 and N294).

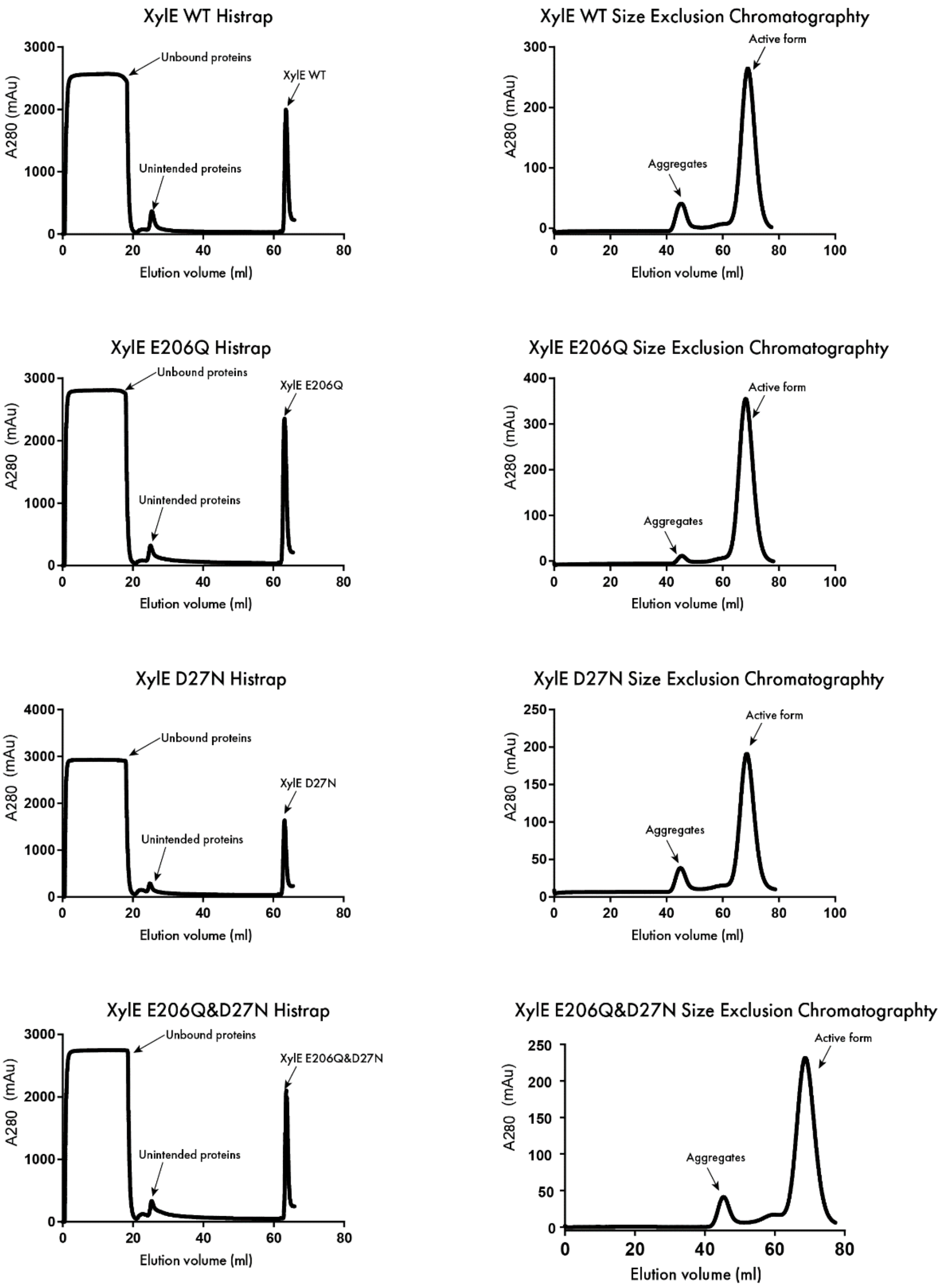

Supplementary Figure 10. Characteristic chromatograms of Histrap and Size Exclusion Chromatography (SEC) of XylE and associated mutants.

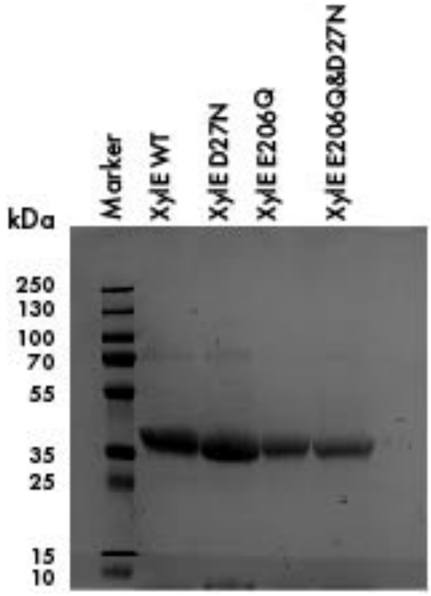

Supplementary Figure 11. SDS-PAGE analysis of XylE WT, D27N, E206Q, and E206Q&D27N. Each purification for XylE wild-type and mutants was repeated at least three times and these characteristic bands correspond to denatured proteins. Proteins were running at the same MW and detected with CBB staining.

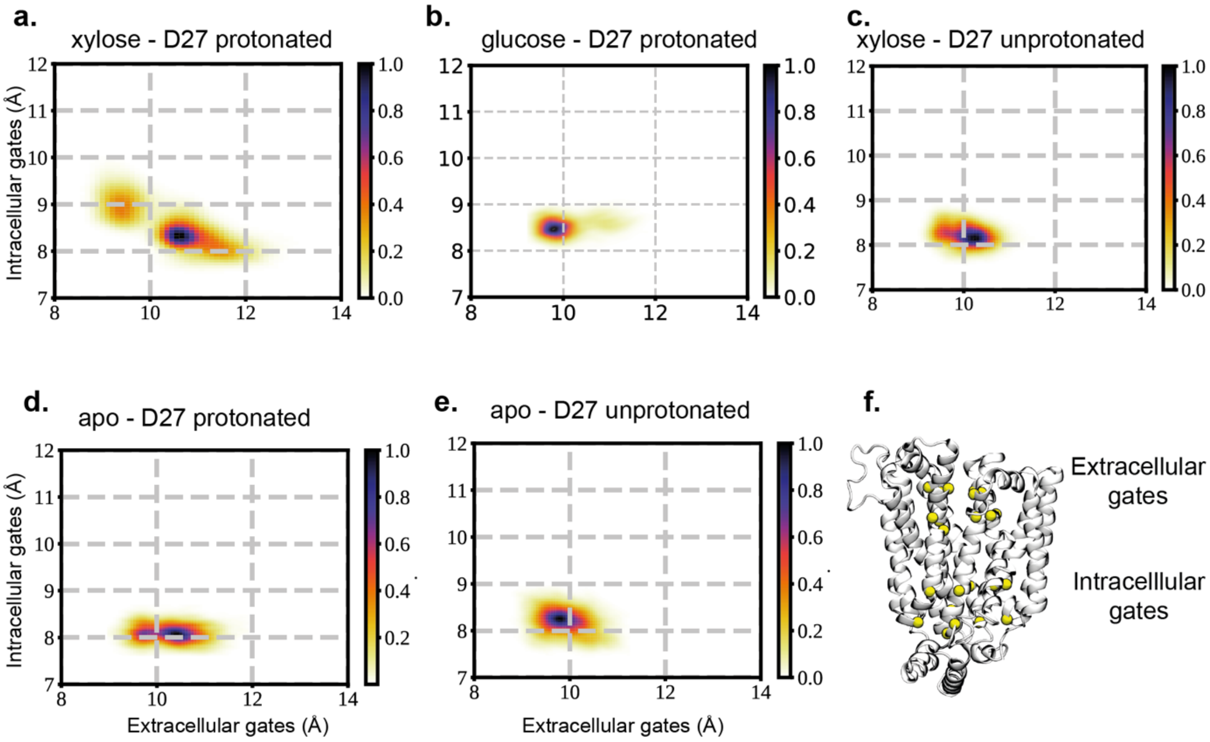

Supplementary Figure 12. Projection of the trajectory on the 2D space spanned by Extracellular and Intracellular gate distances. The intracellular gating distance is defined as the center of mass distance between the two groups of Ca-Ca residues: group 1 (residues 75–80, 149–154, 160–16) and group 2 (residues 332–337, 391–397, 404–410)

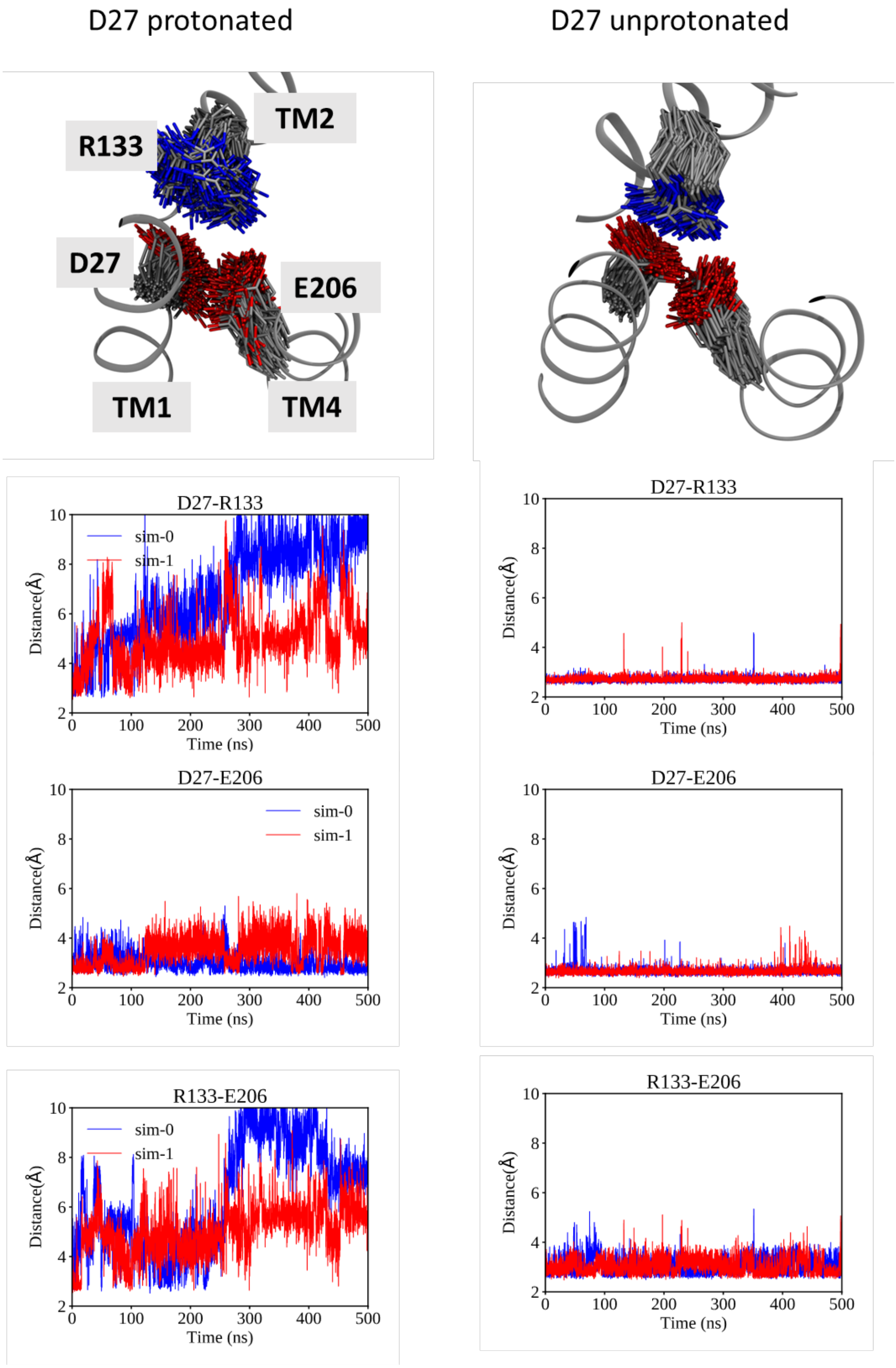

Supplementary Figure 13. Dynamics of the H⁺- Binding Site: Left Panel (D27 Protonated). Upper Left: Conformational sampling of the salt bridging residue (D27, E206 & R133) during the course of simulation for the D27 protonated case. Helices encompassing the salt bridging residues namely TM1, 2 and 4 are depicted as white ribbon. The residues D27, R133 and E206 are shown as sticks colored by atom type sampled every 1ns during the

course of 500 ns MD simulation. Time evolution of the distance between the salt bridging residues in the independent MD simulation for the D27 protonated case depicted by minimum distance between R133:N–D27:O (upper), D27:O–E206:O (middle) and R133:N–E206:O (lower). The distance trace for the two independent MD runs are colored blue and red respectively. Right Panel (D27 unprotonated). Upper Left: Conformational sampling of the salt bridging residue (D27, E206 & R133) during the course of simulation for the D27 unprotonated case. Helices encompassing the salt bridging residues namely TM1, 2 and 4 is depicted as white ribbon. The residues D27, R133 and E206 are shown as sticks colored by atom type sampled every 1ns during the course of 500 ns MD simulation. Time evolution of the distance between the salt bridging residues in the independent MD simulation for the D27 unprotonated case depicted by minimum distance between R133:N–D27:O (upper), D27:O–E206:O (middle) and R133:N–E206:O (lower).

References

1. Martens, C., et al., *Direct protein-lipid interactions shape the conformational landscape of secondary transporters.* Nat Commun, 2018. **9**(1): p. 4151.
2. Li, H., A.D. Robertson, and J.H. Jensen, *Very fast empirical prediction and rationalization of protein pKa values.* Proteins, 2005. **61**(4): p. 704-21.
