# Supplementary material for: Hydrogen-deuterium exchange mass spectrometry captures distinct dynamics upon substrate and inhibitor binding to a transporter
Source: Nat Commun. 2020 Dec 2;11:6162. doi: 10.1038/s41467-020-20032-3 (PMC7710758; doi:10.1038/s41467-020-20032-3)
Supplement: Supplementary file 3 — Supplementary Data 1 [file 41467_2020_20032_MOESM3_ESM.pdf]

Hydrogen-deuterium exchange mass spectrometry captures distinct dynamics upon substrate and inhibitor binding to a transporter

Jia et al.

Supplementary Data File 1

Supplementary data file 1. Tables reporting experimental details for HDX-MS experiments. Data is presented in **Supplementary Figure 3**.

1. ΔHDX = (XylE WT + Xylose) vs (XylE WT)

	XylE WT + Xylose	XylE WT
**HDX reaction details**	10mM potassium phosphate in H_2O pH 7.0, 0.02%DDM	
**HDX time course (min)**	0.5, 5, and 30 minutes	
**Back-exchange (mean/IQR)**	ND	
**Number of peptides**	202	202
**Sequence Coverage**	97.8%	97.8%
**Average peptide length / Redundancy**	9.1 /3.7	9.1 /3.7
**Replicates (biological or technical)**	3 (biological)	3 (biological)
**Repeatability (average SD)**	0.062	0.062
**Significant differences in sum ΔHDX**	CI 99% ± 0.85 Da	

2. ΔHDX = (XylE D27N) vs (XylE WT)

	XylE D27N	XylE WT
**HDX reaction details**	10mM potassium phosphate in H_2O pH 7.0, 0.02%DDM	
**HDX time course (min)**	0.5, 5, and 30 minutes	
**Back-exchange (mean/IQR)**	ND	
**Number of peptides**	301	301
**Sequence Coverage**	100.0%	100.0%
**Average peptide length / Redundancy**	8.6 / 5.3	8.6 / 5.3
**Replicates (biological or technical)**	3 (biological)	3 (biological)
**Repeatability (average SD)**	0.062	0.063
**Significant differences in sum ΔHDX**	CI 99% ± 0.99 Da	

3. ΔHDX = (XylE D27N + Xylose) vs (XylE WT)

	XylE D27N + Xylose	XylE WT
**HDX reaction details**	10mM potassium phosphate in H_2O pH 7.0, 0.02%DDM	
**HDX time course (min)**	0.5, 5, and 30 minutes	
**Back-exchange (mean/IQR)**	ND	
**Number of peptides**	205	205
**Sequence Coverage**	597.8%	97.8%
**Average peptide length / Redundancy**	9.5 / 3.9	9.4 / 3.9
**Replicates (biological or technical)**	3 (biological)	3 (biological)
**Repeatability (average SD)**	0.062	0.062
**Significant differences in sum ΔHDX**	CI 99% ± 0.90 Da	

4. ΔHDX = (XylE E206Q) vs (XylE WT)

	XylE E206Q	XylE WT
**HDX reaction details**	10mM potassium phosphate in H_2O pH 7.0, 0.02%DDM	
**HDX time course (min)**	0.5, 5, and 30 minutes	
**Back-exchange (mean/IQR)**	ND	
**Number of peptides**	183	182
**Sequence Coverage**	97.4%	97.4%
**Average peptide length / Redundancy**	9.2 /3.4	9.1 /3.4
**Replicates (biological or technical)**	3 (biological)	3 (biological)
**Repeatability (average SD)**	0.061	0.061
**Significant differences in sum ΔHDX**	CI 99% ± 0.83 Da	

5. ΔHDX = (XylE E206Q + Xylose) vs (XylE WT)

	XylE E206Q + Xylose	XylE WT
**HDX reaction details**	10mM potassium phosphate in H_2O pH 7.0, 0.02%DDM	
**HDX time course (min)**	0.5, 5, and 30 minutes	
**Back-exchange (mean/IQR)**	ND	
**Number of peptides**	200	202
**Sequence Coverage**	96.9%	97.8%
**Average peptide length / Redundancy**	9.1 / 3.7	9.1 / 3.7
**Replicates (biological or technical)**	3 (biological)	3 (biological)
**Repeatability (average SD)**	0.061	0.061
**Significant differences in sum ΔHDX**	CI 99% ± 0.84 Da	

6. ΔHDX = (XylE E206Q&D27N) vs (XylE WT)

	XylE E206Q&D27N	XylE WT
**HDX reaction details**	10mM potassium phosphate in H_2O pH 7.0, 0.02%DDM	
**HDX time course (min)**	0.5, 5, and 30 minutes	
**Back-exchange (mean/IQR)**	ND	
**Number of peptides**	263	259
**Sequence Coverage**	99.0%	99.0%
**Average peptide length / Redundancy**	9.0 / 4.8	9.0 / 4.7
**Replicates (biological or technical)**	3 (biological)	3 (biological)
**Repeatability (average SD)**	0.075	0.071
**Significant differences in sum ΔHDX**	CI 99% ± 1.06 Da	

7. ΔHDX = (XylE E206Q&D27N + Xylose) vs (XylE WT)

	XylE E206Q&D27N + Xylose	XylE WT
**HDX reaction details**	10mM potassium phosphate in H_2O pH 7.0, 0.02%DDM	
**HDX time course (min)**	0.5, 5, and 30 minutes	
**Back-exchange (mean/IQR)**	ND	
**Number of peptides**	205	205
**Sequence Coverage**	97.8%	97.8%
**Average peptide length / Redundancy**	9.5 / 4.0	9.4 / 3.9
**Replicates (biological or technical)**	3 (biological)	3 (biological)
**Repeatability (average SD)**	0.061	0.062
**Significant differences in sum ΔHDX**	CI 99% ± 0.89 Da	

8. ΔHDX = (XylE WT + Xylose) vs (XylE D27N)

	XylE WT + Xylose	XylE D27N
**HDX reaction details**	10mM potassium phosphate in H_2O pH 7.0, 0.02%DDM	
**HDX time course (min)**	0.5, 5, and 30 minutes	
**Back-exchange (mean/IQR)**	ND	
**Number of peptides**	250	250
**Sequence Coverage**	96.5%	96.5%
**Average peptide length / Redundancy**	9.0 / 4.6	9.0 / 4.6
**Replicates (biological or technical)**	3 (biological)	3 (biological)
**Repeatability (average SD)**	0.062	0.061
**Significant differences in sum ΔHDX**	CI 99% ± 0.86 Da	

9. ΔHDX = (XylE D27N + Xylose) vs (XylE WT+Xylose)

	XylE D27N + Xylose	XylE WT+Xylose
**HDX reaction details**	10mM potassium phosphate in H_2O pH 7.0, 0.02%DDM	
**HDX time course (min)**	0.5, 5, and 30 minutes	
**Back-exchange (mean/IQR)**	ND	
**Number of peptides**	266	269
**Sequence Coverage**	94.3%	94.3%
**Average peptide length / Redundancy**	8.8 / 4.8	8.9 / 4.9
**Replicates (biological or technical)**	3 (biological)	3 (biological)
**Repeatability (average SD)**	0.056	0.062
**Significant differences in sum ΔHDX**	CI 99% ± 0.84 Da	

10. ΔHDX = (XylE E206) vs (XylE WT + Xylose)

	XylE E206	XylE WT + Xylose
**HDX reaction details**	10mM potassium phosphate in H_2O pH 7.0, 0.02%DDM	
**HDX time course (min)**	0.5, 5, and 30 minutes	
**Back-exchange (mean/IQR)**	ND	
**Number of peptides**	204	205
**Sequence Coverage**	98.0%	98.0%
**Average peptide length / Redundancy**	9.0 / 3.7	9.0 / 3.8
**Replicates (biological or technical)**	3 (biological)	3 (biological)
**Repeatability (average SD)**	0.069	0.070
**Significant differences in sum ΔHDX**	CI 99% ± 0.97 Da	

11. ΔHDX = (XylE E206Q + Xylose) vs (XylE WT + Xylose)

	XylE E206Q + Xylose	XylE WT + Xylose
**HDX reaction details**	10mM potassium phosphate in H_2O pH 7.0, 0.02%DDM	
**HDX time course (min)**	0.5, 5, and 30 minutes	
**Back-exchange (mean/IQR)**	ND	
**Number of peptides**	268	269
**Sequence Coverage**	94.3%	94.3%
**Average peptide length / Redundancy**	8.9 / 4.8	8.9 / 4.9
**Replicates (biological or technical)**	3 (biological)	3 (biological)
**Repeatability (average SD)**	0.060	0.062
**Significant differences in sum ΔHDX**	CI 99% ± 0.86 Da	

12. ΔHDX = (XylE E206Q&D27N) vs (XylE WT + Xylose)

	XylE E206Q&D27N	XylE WT + Xylose
**HDX reaction details**	10mM potassium phosphate in H_2O pH 7.0, 0.02%DDM	
**HDX time course (min)**	0.5, 5, and 30 minutes	
**Back-exchange (mean/IQR)**	ND	
**Number of peptides**	202	202
**Sequence Coverage**	97.8%	97.8%
**Average peptide length / Redundancy**	9.1 / 3.7	9.1 / 3.7
**Replicates (biological or technical)**	3 (biological)	3 (biological)
**Repeatability (average SD)**	0.068	0.068
**Significant differences in sum ΔHDX**	CI 99% ± 1.06 Da	

13. ΔHDX = (XylE E206Q&D27N + Xylose) vs (XylE WT + Xylose)

	XylE E206Q&D27N + Xylose	XylE WT + Xylose
**HDX reaction details**	10mM potassium phosphate in H_2O pH 7.0, 0.02%DDM	
**HDX time course (min)**	0.5, 5, and 30 minutes	
**Back-exchange (mean/IQR)**	ND	
**Number of peptides**	251	250
**Sequence Coverage**	96.5%	96.5%
**Average peptide length / Redundancy**	9.0 / 4.6	9.0 / 4.6
**Replicates (biological or technical)**	3 (biological)	3 (biological)
**Repeatability (average SD)**	0.064	0.062
**Significant differences in sum ΔHDX**	CI 99% ± 0.87 Da	

14. ΔHDX = (XylE D27N+ Xylose) vs (XylE D27N)

	XylE D27N+ Xylose	XylE D27N
**HDX reaction details**	10mM potassium phosphate in H_2O pH 7.0, 0.02%DDM	
**HDX time course (min)**	0.5, 5, and 30 minutes	
**Back-exchange (mean/IQR)**	ND	
**Number of peptides**	205	203
**Sequence Coverage**	97.8%	96.7%
**Average peptide length / Redundancy**	9.5 / 3.9	9.4 / 3.9
**Replicates (biological or technical)**	3 (biological)	3 (biological)
**Repeatability (average SD)**	0.062	0.067
**Significant differences in sum ΔHDX**	CI 99% ± 0.92 Da	

15. ΔHDX = (XylE E206Q) vs (XylE D27N)

	XylE E206Q	XylE D27N
**HDX reaction details**	10mM potassium phosphate in H_2O pH 7.0, 0.02%DDM	
**HDX time course (min)**	0.5, 5, and 30 minutes	
**Back-exchange (mean/IQR)**	ND	
**Number of peptides**	183	182
**Sequence Coverage**	97.4%	97.4%
**Average peptide length / Redundancy**	9.2 / 3.4	9.1 / 3.4
**Replicates (biological or technical)**	3 (biological)	3 (biological)
**Repeatability (average SD)**	0.061	0.064
**Significant differences in sum ΔHDX**	CI 99% ± 0.86 Da	

16. ΔHDX = (XylE E206Q + Xylose) vs (XylE D27N)

	XylE E206Q + Xylose	XylE D27N
**HDX reaction details**	10mM potassium phosphate in H_2O pH 7.0, 0.02%DDM	
**HDX time course (min)**	0.5, 5, and 30 minutes	
**Back-exchange (mean/IQR)**	ND	
**Number of peptides**	251	250
**Sequence Coverage**	96.5%	96.5%
**Average peptide length / Redundancy**	9.0 / 4.6	9.0 / 4.6
**Replicates (biological or technical)**	3 (biological)	3 (biological)
**Repeatability (average SD)**	0.059	0.061
**Significant differences in sum ΔHDX**	CI 99% ± 0.84 Da	

17. ΔHDX = (XylE E206Q&D27N) vs (XylE D27N)

	XylE E206Q&D27N	XylE D27N
**HDX reaction details**	10mM potassium phosphate in H_2O pH 7.0, 0.02%DDM	
**HDX time course (min)**	0.5, 5, and 30 minutes	
**Back-exchange (mean/IQR)**	ND	
**Number of peptides**	243	242
**Sequence Coverage**	98.6%	98.6%
**Average peptide length / Redundancy**	9.2 / 4.6	9.2 / 4.5
**Replicates (biological or technical)**	3 (biological)	3 (biological)
**Repeatability (average SD)**	0.065	0.063
**Significant differences in sum ΔHDX**	CI 99% ± 0.90 Da	

18. ΔHDX = (XylE E206Q&D27N + Xylose) vs (XylE D27N)

	XylE E206Q&D27N + Xylose	XylE D27N
**HDX reaction details**	10mM potassium phosphate in H_2O pH 7.0, 0.02%DDM	
**HDX time course (min)**	0.5, 5, and 30 minutes	
**Back-exchange (mean/IQR)**	ND	
**Number of peptides**	205	203
**Sequence Coverage**	97.8%	96.7%
**Average peptide length / Redundancy**	9.5 / 4.0	9.4 / 3.9
**Replicates (biological or technical)**	3 (biological)	3 (biological)
**Repeatability (average SD)**	0.061	0.067
**Significant differences in sum ΔHDX**	CI 99% ± 0.92 Da	

19. ΔHDX = (XylE D27N + Xylose) vs (XylE E206Q)

	XylE D27N + Xylose	XylE E206Q
**HDX reaction details**	10mM potassium phosphate in H_2O pH 7.0, 0.02%DDM	
**HDX time course (min)**	0.5, 5, and 30 minutes	
**Back-exchange (mean/IQR)**	ND	
**Number of peptides**	205	206
**Sequence Coverage**	97.8%	97.8%
**Average peptide length / Redundancy**	9.5 / 3.9	9.6 / 4.0
**Replicates (biological or technical)**	3 (biological)	3 (biological)
**Repeatability (average SD)**	0.062	0.070
**Significant differences in sum ΔHDX**	CI 99% ± 0.94 Da	

20. ΔHDX = (XylE E206Q + Xylose) vs (XylE D27N+Xylose)

	XylE E206Q + Xylose	XylE D27N+Xylose
**HDX reaction details**	10mM potassium phosphate in H_2O pH 7.0, 0.02%DDM	
**HDX time course (min)**	0.5, 5, and 30 minutes	
**Back-exchange (mean/IQR)**	ND	
**Number of peptides**	252	256
**Sequence Coverage**	93.9%	93.9%
**Average peptide length / Redundancy**	8.9 / 4.5	8.9 / 4.7
**Replicates (biological or technical)**	3 (biological)	3 (biological)
**Repeatability (average SD)**	0.062	0.061
**Significant differences in sum ΔHDX**	CI 99% ± 0.90 Da	

21. ΔHDX = (XylE E206Q&D27N) vs (XylE D27N + Xylose)

	XylE E206Q&D27N	XylE D27N + Xylose
**HDX reaction details**	10mM potassium phosphate in H_2O pH 7.0, 0.02%DDM	
**HDX time course (min)**	0.5, 5, and 30 minutes	
**Back-exchange (mean/IQR)**	ND	
**Number of peptides**	230	231
**Sequence Coverage**	99.0%	99.0%
**Average peptide length / Redundancy**	8.9 / 4.2	9.0 / 4.2
**Replicates (biological or technical)**	3 (biological)	3 (biological)
**Repeatability (average SD)**	0.057	0.059
**Significant differences in sum ΔHDX**	CI 99% ± 0.81Da	

22. ΔHDX = (XylE E206Q&D27N + Xylose) vs (XylE D27N + Xylose)

	XylE E206Q&D27N + Xylose	XylE D27N + Xylose
**HDX reaction details**	10mM potassium phosphate in H_2O pH 7.0, 0.02%DDM	
**HDX time course (min)**	0.5, 5, and 30 minutes	
**Back-exchange (mean/IQR)**	ND	
**Number of peptides**	205	205
**Sequence Coverage**	97.8%	97.8%
**Average peptide length / Redundancy**	9.5 / 3.9	9.5 / 4.0
**Replicates (biological or technical)**	3 (biological)	3 (biological)
**Repeatability (average SD)**	0.061	0.062
**Significant differences in sum ΔHDX**	CI 99% ± 0.89 Da	

23. ΔHDX = (XylE E206Q + Xylose) vs (XylE E206Q)

	XylE E206Q + Xylose	XylE E206Q
**HDX reaction details**	10mM potassium phosphate in H_2O pH 7.0, 0.02%DDM	
**HDX time course (min)**	0.5, 5, and 30 minutes	
**Back-exchange (mean/IQR)**	ND	
**Number of peptides**	222	220
**Sequence Coverage**	98.0%	97.4%
**Average peptide length / Redundancy**	9.2 / 4.1	9.2 / 4.2
**Replicates (biological or technical)**	3 (biological)	3 (biological)
**Repeatability (average SD)**	0.062	0.066
**Significant differences in sum ΔHDX**	CI 99% ± 0.90 Da	

24. ΔHDX = (XylE E206Q&D27N) vs (XylE E206Q)

	XylE E206Q&D27N	XylE E206Q
**HDX reaction details**	10mM potassium phosphate in H_2O pH 7.0, 0.02%DDM	
**HDX time course (min)**	0.5, 5, and 30 minutes	
**Back-exchange (mean/IQR)**	ND	
**Number of peptides**	221	220
**Sequence Coverage**	97.4%	97.4%
**Average peptide length / Redundancy**	9.2 / 4.1	9.2 / 4.1
**Replicates (biological or technical)**	3 (biological)	3 (biological)
**Repeatability (average SD)**	0.064	0.066
**Significant differences in sum ΔHDX**	CI 99% ± 0.92 Da	

25. ΔHDX = (XylE E206Q&D27N + Xylose) vs (XylE E206Q)

	XylE E206Q&D27N + Xylose	XylE E206Q
**HDX reaction details**	10mM potassium phosphate in H_2O pH 7.0, 0.02%DDM	
**HDX time course (min)**	0.5, 5, and 30 minutes	
**Back-exchange (mean/IQR)**	ND	
**Number of peptides**	205	206
**Sequence Coverage**	97.8%	97.8%
**Average peptide length / Redundancy**	9.5 / 4.0	9.6 / 4.0
**Replicates (biological or technical)**	3 (biological)	3 (biological)
**Repeatability (average SD)**	0.061	0.070
**Significant differences in sum ΔHDX**	CI 99% ± 0.94 Da	

26. ΔHDX = (XylE E206Q&D27N) vs (XylE E206Q +Xylose)

	XylE E206Q&D27N	XylE E206Q +Xylose
**HDX reaction details**	10mM potassium phosphate in H_2O pH 7.0, 0.02%DDM	
**HDX time course (min)**	0.5, 5, and 30 minutes	
**Back-exchange (mean/IQR)**	ND	
**Number of peptides**	202	200
**Sequence Coverage**	97.8%	96.9%
**Average peptide length / Redundancy**	9.1 / 3.7	9.1 / 3.7
**Replicates (biological or technical)**	3 (biological)	3 (biological)
**Repeatability (average SD)**	0.068	0.061
**Significant differences in sum ΔHDX**	CI 99% ± 0.89 Da	

27. ΔHDX = (XylE E206Q&D27N + Xylose) vs (XylE E206Q + Xylose)

	XylE E206Q&D27N + Xylose	XylE E206Q + Xylose
**HDX reaction details**	10mM potassium phosphate in H_2O pH 7.0, 0.02%DDM	
**HDX time course (min)**	0.5, 5, and 30 minutes	
**Back-exchange (mean/IQR)**	ND	
**Number of peptides**	201	200
**Sequence Coverage**	97.8%	96.9%
**Average peptide length / Redundancy**	9.1 / 3.7	9.1 / 3.7
**Replicates (biological or technical)**	3 (biological)	3 (biological)
**Repeatability (average SD)**	0.059	0.061
**Significant differences in sum ΔHDX**	CI 99% ± 0.83 Da	

28. ΔHDX = (XylE E206Q&D27N + Xylose) vs (XylE E206Q&D27N)

	XylE E206Q&D27N + Xylose	XylE E206Q&D27N
**HDX reaction details**	10mM potassium phosphate in H_2O pH 7.0, 0.02%DDM	
**HDX time course (min)**	0.5, 5, and 30 minutes	
**Back-exchange (mean/IQR)**	ND	
**Number of peptides**	201	202
**Sequence Coverage**	97.8%	97.8%
**Average peptide length / Redundancy**	9.1 / 3.7	9.1 / 3.7
**Replicates (biological or technical)**	3 (biological)	3 (biological)
**Repeatability (average SD)**	0.059	0.068
**Significant differences in sum ΔHDX**	CI 99% ± 0.88 Da	

29. ΔHDX = (XylE WT + Glucose) vs (XylE WT)

	XylE WT + Glucose	XylE WT
**HDX reaction details**	10mM potassium phosphate in H_2O pH 7.0, 0.02%DDM	
**HDX time course (min)**	0.5, 5, and 30 minutes	
**Back-exchange (mean/IQR)**	ND	
**Number of peptides**	124	120
**Sequence Coverage**	80.4%	78.2%
**Average peptide length / Redundancy**	8.5 / 2.1	8.3 / 2.0
**Replicates (biological or technical)**	3 (biological)	3 (biological)
**Repeatability (average SD)**	0.055	0.061
**Significant differences in sum ΔHDX**	CI 99% ± 0.77 Da	

30. ΔHDX = (XylE D27N + Glucose) vs (XylE WT)

	XylE D27N + Glucose	XylE WT
**HDX reaction details**	10mM potassium phosphate in H_2O pH 7.0, 0.02%DDM	
**HDX time course (min)**	0.5, 5, and 30 minutes	
**Back-exchange (mean/IQR)**	ND	
**Number of peptides**	124	120
**Sequence Coverage**	80.4%	78.2%
**Average peptide length / Redundancy**	8.5 / 2.1	8.3 / 2.0
**Replicates (biological or technical)**	3 (biological)	3 (biological)
**Repeatability (average SD)**	0.061	0.061
**Significant differences in sum ΔHDX**	CI 99% ± 0.82 Da	

31. ΔHDX = (XylE D27N + Glucose) vs (XylE D27N)

	XylE D27N + Glucose	XylE D27N
**HDX reaction details**	10mM potassium phosphate in H_2O pH 7.0, 0.02%DDM	
**HDX time course (min)**	0.5, 5, and 30 minutes	
**Back-exchange (mean/IQR)**	ND	
**Number of peptides**	124	120
**Sequence Coverage**	80.5%	80.5%
**Average peptide length / Redundancy**	8.5 / 2.1	8.5 / 2.1
**Replicates (biological or technical)**	3 (biological)	3 (biological)
**Repeatability (average SD)**	0.061	0.060
**Significant differences in sum ΔHDX**	CI 99% ± 0.78 Da	

32. ΔHDX = (XylE D27N + Glucose) vs (XylE WT + Glucose)

	XylE D27N + Glucose	XylE WT + Xylose
**HDX reaction details**	10mM potassium phosphate in H_2O pH 7.0, 0.02%DDM	
**HDX time course (min)**	0.5, 5, and 30 minutes	
**Back-exchange (mean/IQR)**	ND	
**Number of peptides**	181	178
**Sequence Coverage**	92.7%	90.8%
**Average peptide length / Redundancy**	9.2 / 3.4	9.1 / 3.3
**Replicates (biological or technical)**	3 (biological)	3 (biological)
**Repeatability (average SD)**	0.057	0.060
**Significant differences in sum ΔHDX**	CI 99% ± 0.78 Da	
