# Supplementary material for: Hydrogen-deuterium exchange mass spectrometry captures distinct dynamics upon substrate and inhibitor binding to a transporter
Source: Nat Commun. 2020 Dec 2;11:6162. doi: 10.1038/s41467-020-20032-3 (PMC7710758; doi:10.1038/s41467-020-20032-3)
Supplement: Supplementary file 4 — Reporting Summary [file 41467_2020_20032_MOESM4_ESM.pdf]

nature research

Corresponding author(s): Argyris Politis, Paula Booth, Chloe Martens

Last updated by author(s): Oct 7, 2020

Reporting Summary

Nature Research wishes to improve the reproducibility of the work that we publish. This form provides structure for consistency and transparency in reporting. For further information on Nature Research policies, see Authors & Referees and the Editorial Policy Checklist.

Statistics

For all statistical analyses, confirm that the following items are present in the figure legend, table legend, main text, or Methods section.

n/a	Confirmed	
☐	☒	The exact sample size (n) for each experimental group/condition, given as a discrete number and unit of measurement
☐	☒	A statement on whether measurements were taken from distinct samples or whether the same sample was measured repeatedly
☒	☐	The statistical test(s) used AND whether they are one- or two-sided<br>*Only common tests should be described solely by name; describe more complex techniques in the Methods section.*
☒	☐	A description of all covariates tested
☒	☐	A description of any assumptions or corrections, such as tests of normality and adjustment for multiple comparisons
☐	☒	A full description of the statistical parameters including central tendency (e.g. means) or other basic estimates (e.g. regression coefficient) AND variation (e.g. standard deviation) or associated estimates of uncertainty (e.g. confidence intervals)
☒	☐	For null hypothesis testing, the test statistic (e.g. F, t, r) with confidence intervals, effect sizes, degrees of freedom and P value noted<br>*Give P values as exact values whenever suitable.*
☒	☐	For Bayesian analysis, information on the choice of priors and Markov chain Monte Carlo settings
☒	☐	For hierarchical and complex designs, identification of the appropriate level for tests and full reporting of outcomes
☒	☐	Estimates of effect sizes (e.g. Cohen's d, Pearson's r), indicating how they were calculated

Our web collection on statistics for biologists contains articles on many of the points above.

Software and code

Policy information about availability of computer code

Data collection	Raw MS data were analysed using Waters Protein Lynx Global Server (version 2.5.1; PLGS) and DynamX (version 3.0) to yield tabulated data that were given to Deuteros (version 1 and 2) for analysis.
Data analysis	Deuteros (versions 1 and 2) was used for the analysis of HDX-MS data (available at https://github.com/andymlau/Deuteros)

For manuscripts utilizing custom algorithms or software that are central to the research but not yet described in published literature, software must be made available to editors/reviewers. We strongly encourage code deposition in a community repository (e.g. GitHub). See the Nature Research guidelines for submitting code & software for further information.

Data

Policy information about availability of data

All manuscripts must include a data availability statement. This statement should provide the following information, where applicable:

- Accession codes, unique identifiers, or web links for publicly available datasets
- A list of figures that have associated raw data
- A description of any restrictions on data availability

Data supporting the findings of this paper are available from corresponding author upon reasonable request. All the deuterium uptake plots of the experiments presented for XylE are available on figshare data repository using the following link: https://figshare.com/s/52d498fe3b10c60b64a4. Moreover, spectrometry proteomics data have been deposited to the ProteomeXchange Consortium via the PRIDE partner repository with the dataset identifier PXD018145.

Field-specific reporting

Please select the one below that is the best fit for your research. If you are not sure, read the appropriate sections before making your selection.

☒ Life sciences ☐ Behavioural & social sciences ☐ Ecological, evolutionary & environmental sciences

For a reference copy of the document with all sections, see nature.com/documents/nr-reporting-summary-flat.pdf

Life sciences study design

All studies must disclose on these points even when the disclosure is negative.

Sample size	HDX data were collected as biological and technical triplicates, following community suggestions detailed in Masson et al. 2019. Nature Methods, 16, 595-602. All experimental procedures were inherited from Martens et al., 2018. Nature Commun,9(1): p. 4151 and have been shown to yield a high number of high quality peptides for this study (~150 peptides).
Data exclusions	There are two points of data exclusion in this study. The first, concerns the removal of low quality peptides during manual curation of raw MS data in DynamX (version 3.0) software. Ions which cannot be assigned in DynamX due to low signal intensity or show inconsistent m/z or isotopic envelopes between replicates are removed from further analysis. For any peptide, data belonging to only one charge state is retained (typically selecting the charge state with the highest signal intensity or by selecting the most completely assigned isotopic envelope) and all other charge states are deemed as redundant and removed. Second, any peptides including the site of mutation are excluded from any downstream analysis.
Replication	HDX data were collected as biological and technical triplicates, following community suggestions detailed in Masson et al. 2019. Nature Methods, 16, 595-602. Measurements were performed in triplicate for each labelling timepoint. All data presented for differential HDX-MS comparisons were successfully acquired on the same day to minimize instrumental variations.
Randomization	No randomization was performed for this study.
Blinding	No blinding was performed for this study.

Reporting for specific materials, systems and methods

We require information from authors about some types of materials, experimental systems and methods used in many studies. Here, indicate whether each material, system or method listed is relevant to your study. If you are not sure if a list item applies to your research, read the appropriate section before selecting a response.

Materials & experimental systems

n/a	Involved in the study
☒	☐ Antibodies
☒	☐ Eukaryotic cell lines
☒	☐ Palaeontology
☒	☐ Animals and other organisms
☒	☐ Human research participants
☒	☐ Clinical data

Methods

n/a	Involved in the study
☒	☐ ChIP-seq
☒	☐ Flow cytometry
☒	☐ MRI-based neuroimaging
